# Fabrication of Interface Engineered S‐Scheme Heterojunction Nanocatalyst for Ultrasound‐Triggered Sustainable Cancer Therapy

**DOI:** 10.1002/advs.202308546

**Published:** 2024-02-11

**Authors:** Meng Yuan, Ling Yang, Zhuang Yang, Zhizi Ma, Jie Ma, Zhendong Liu, Ping'an Ma, Ziyong Cheng, Aziz Maleki, Jun Lin

**Affiliations:** ^1^ State Key Laboratory of Rare Earth Resource Utilization Changchun Institute of Applied Chemistry, Chinese Academy of Sciences Changchun 130022 China; ^2^ School of Applied Chemistry and Engineering University of Science and Technology of China Hefei 230026 China; ^3^ Key Laboratory of Superlight Materials and Surface Technology Ministry of Education College of Materials Science and Chemical Engineering Harbin Engineering University Harbin 150001 China; ^4^ Zanjan Pharmaceutical Nanotechnology Research Center (ZPNRC) and Department of Pharmaceutical Nanotechnology (School of pharmacy) Zanjan University of Medical Sciences Zanjan 4513956184 Iran

**Keywords:** CO therapy, SDT, sonocatalysis, S‐scheme heterojunction

## Abstract

In order to establish a set of perfect heterojunction designs and characterization schemes, step‐scheme (S‐scheme) BiOBr@Bi_2_S_3_ nanoheterojunctions that enable the charge separation and expand the scope of catalytic reactions, aiming to promote the development and improvement of heterojunction engineering is developed. In this kind of heterojunction system, the Fermi levels mediate the formation of the internal electric field at the interface and guide the recombination of the weak redox carriers, while the strong redox carriers are retained. Thus, these high‐energy electrons and holes are able to catalyze a variety of substrates in the tumor microenvironment, such as the reduction of oxygen and carbon dioxide to superoxide radicals and carbon monoxide (CO), and the oxidation of H_2_O to hydroxyl radicals, thus achieving sonodynamic therapy and CO combined therapy. Mechanistically, the generated reactive oxygen species and CO damage DNA and inhibit cancer cell energy levels, respectively, to synergistically induce tumor cell apoptosis. This study provides new insights into the realization of high efficiency and low toxicity in catalytic therapy from a unique perspective of materials design. It is anticipated that this catalytic therapeutic method will garner significant interest in the sonocatalytic nanomedicine field.

## Graphical abstract

An interface‐engineered S‐scheme heterojunction based on BiOBr@Bi_2_S_3_ nanocomposite is developed to prolong the life span of sonoexcited electrons and holes, expand the selectivity of catalytic substrates and therapeutic products, and promote tumor catalytic therapy. Moreover, its proof‐of‐concept application becomes a new SDT and CO combination therapy platform based on heterojunction.

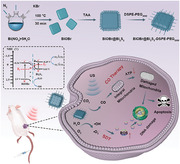

## 1 Introduction

In the past several years, many nanocatalysts such as nanozymes,^[^ ^1^ ^]^ photocatalysts,^[^ ^2^ ^]^ sonocatalysts,^[^ ^3^ ^]^ and electrocatalysts,^[^ ^4^ ^]^ have been put into use in vivo to initiate catalytic reactions and improve the biological microenvironment for eliciting therapeutic effects. This tendency has led to rapid developments of nanocatalytic medicine^[^ ^5^ ^]^ and offers effective therapeutic options for various common diseases, such as cancer,^[^ ^6^ ^]^ bacterial infections,^[^ ^7^ ^]^ and inflammation.^[^ ^8^ ^]^ Among them, exogenous stimulation [e.g., light, ultrasound (US), electricity]‐assisted catalysis has become the main strategy of disease treatment because of its higher operability.^[^ ^9^ ^]^ From the perspective of the catalytic reaction mechanism, the crucial factor determining the catalytic efficiency of such emerging catalytic therapeutic modality is the separation efficiency of electron‐hole pairs. A defect engineering strategy is commonly used to improve the efficiency of single‐component catalyst charge separation.^[^ ^10^ ^]^ The introduction of vacancies or doping elements can not only reduce the bandgap of the nanocatalysts but also trap the excited holes/electrons to promote charge separation.^[^ ^11^ ^]^ Although the narrow bandgap nanocatalysts can promote charge separation, their low redox potential limits the range of therapeutic products. However, nanocatalysts with strong redox capacity must have more positive valence band (VB) potential and more negative conduction band (CB) potential, that is, wide bandgap, which inevitably leads to difficult charge separation and unsatisfactory catalytic performance. Therefore, it is difficult to meet the requirements of high catalytic activity and various catalytic products simultaneously for single‐component catalysts.

The method of constructing heterojunction by coupling two semiconductors brings hope to break this contradiction.^[^ ^12^ ^]^ Among them, type‐II and Z‐scheme heterojunction have been widely concerned because of their excellent charge separation ability.^[^ ^13^ ^]^ While the charge‐transfer paths of these two heterojunctions seem to be perfect at separating charge, there are still some shortcomings upon closer examination.^[^ ^14^ ^]^ In the type‐II heterojunction, electrons move from a high position to a low position and holes move from a low position to a high position. From a thermodynamic point of view, charge separation is at the cost of reducing the redox capacity of semiconductors. This is consistent with single‐component catalysts, which are not conducive to the occurrence of catalytic reactions. In addition, the repulsion between electrons (or holes) prevents sustained charge transfer. Therefore, it can be seen that the type‐II charge transfer route is unfavorable. To solve these problems of type‐II heterojunction, the concept of Z‐scheme heterojunction is proposed.^[^ ^15^ ^]^ Z‐scheme heterojunctions are mainly divided into three types: 1) Traditional Z‐scheme heterojunctions using redox ion pairs as charge transport mediators. 2) All‐solid‐state Z‐scheme heterojunctions with a solid conductor as a carrier transfer intermediate. 3) Direct Z‐scheme heterojunction without any charge transfer medium. Unfortunately, for the first two types of Z‐scheme heterojunctions, charge transfer cannot occur in the desired way, and other possible charge transfer paths may dominate. For example, the effective excited state electrons and holes are consumed, while the excited electrons and holes with weak redox capacity are retained. For direct Z‐scheme heterojunctions, the charge transfer mechanism is fuzzy. To solve the above problems and mistakes, a new step‐scheme (S‐scheme) heterojunction concept is proposed on the basis of direct Z‐scheme heterojunction to promote the development of heterojunction engineering.^[^ ^16^ ^]^ This S‐scheme heterojunction gets its name because the electron transfer route resembles a step. In general, the S‐scheme heterojunction consists of a reduction catalyst and an oxidation catalyst. The reduction catalyst has higher CB and VB positions and lower work function (*W*) than the oxidation catalyst. When the reduction catalyst and the oxidation catalyst come into contact, the electrons at the interface in the reduction catalyst will voluntarily transfer to the oxidation catalyst, which causes the Fermi level (*E_f_*) to bend in the interface region. At the same time, the directed migration of free electrons leads to band bending and the formation of an internal electric field (IEF) at the interface. Under the external stimulus, both two catalysts are activated simultaneously, and the holes on the VB of the reduction catalyst recombine with the electrons on the CB of the oxidation catalyst facilitated by the energy band bending and IEF. Meanwhile, the holes and electrons with strong redox ability are retained to participate in the oxidation reaction and reduction reaction, respectively. In summary, S‐scheme heterojunction offers three key advantages that determine its application prospects in the biomedical field, namely, 1) high charge separation efficiency, 2) strong redox ability, and 3) a wide range of catalytic reactions.

**Scheme 1:**
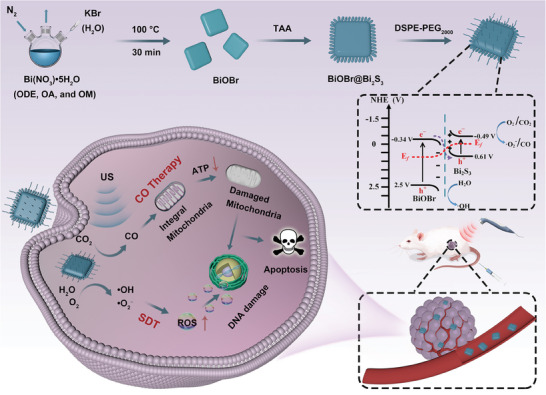
Schematic illustration of synthesis and antitumor therapy of BiOBr@Bi_2_S_3_‐DSPE‐PEG_2000_.

Based on the above analysis, the charge transfer mechanism of the S‐scheme is superior to that of Z‐scheme. The direct Z‐scheme charge transfer mechanism usually means that the CB of the oxidation catalyst and the VB of the reduction catalyst are very close so that the electron and hole recombination directly occurs when the two contact. However, S‐scheme charge transfer is driven by the difference in *E_f_* between the two semiconductors. However, the design of S‐scheme heterojunction is still in its infancy, which has led to confusion or uncertainty about the concepts of direct Z‐scheme and S‐scheme in most research on heterojunction engineering.^[^ ^17^ ^]^ This situation is detrimental to the application of heterojunction engineering in the treatment of various diseases. It is worth mentioning that the characterization and determination of the charge transfer mechanism is also vital because it is the key to clarifying the structure‐activity relationship. Regrettably, this crucial point has been often neglected in the previously reported studies.^[^ ^18^ ^]^ For example, Chen et al. constructed a 2D heterojunction BiOCl/Bi_2_O_3_ for US‐induced cancer therapy.^[^ ^19^ ^]^ They successfully demonstrated that heterojunction design could not only delay charge recombination but also expand the range of catalytic reactions. However, their study did not distinguish the concepts of Z‐scheme and S‐scheme heterojunction, and there was no direct evidence of a charge transfer route. Therefore, our design concept is to construct catalysts with clear heterostructures and to characterize the types of heterojunctions and charge transfer pathways in detail to prove the successful construction of S‐scheme heterojunctions. We hope that our study can provide a clear idea for the construction and characterization of heterojunctions and promote the development of heterojunction engineering in the biomedical field. We referred to the S‐scheme heterostructures (BiOBr@Bi_2_S_3_) reported in the field of photocatalysis and optimized the synthesis route to prepare the heterostructures suitable for biological applications.^[^ ^20^ ^]^ With regard to heterojunction characterization, the S‐scheme was determined by the characterization of band structures, *W*, and *E_f_* of BiOBr and Bi_2_S_3_. At the same time, the ex situ/in situ irradiated X‐ray photoelectron spectroscopy (XPS) characterization provided direct evidence for the charge transfer mechanism of the S‐scheme. A series of experiments also confirmed that BiOBr@Bi_2_S_3_ S‐scheme heterojunction could enable the effective separation of electrons and holes in space and maintain high reduction/oxidation capacity, respectively. Importantly, two catalytic active sites located at the CB of Bi_2_S_3_ (−0.49 V) and the VB of BiOBr (2.5 V) not only catalyze the generation of conventional ROS to achieve sonodynamic therapy (SDT), such as the reduction of oxygen (O_2_) to superoxide radicals (•O_2_^−^) and the oxidation of H_2_O to hydroxyl radicals (•OH), but also have ability to reduce carbon dioxide (CO_2_) to produce carbon monoxide (CO) for CO therapy (**Scheme** **1**). Although the SDT/CO combination therapy could also be achieved by using the US as an exogenous‐focused energy field to trigger the CO release by CO‐releasing molecules (CORMs), the amount of CO released was limited and the rate was slow, resulting in unsatisfactory therapeutic effect.^[^ ^21^ ^]^ In contrast, the strategy of catalysis‐mediated CO_2_ reduction can not only achieve long‐lasting CO production but also control the site‐specific generation of CO to avoid the risk of blood poisoning. This synchronous production of ROS and CO triggered by remotely controlled US can synergistically inhibit tumor growth and proliferation.

## 2 Results and Discussion

**Figure 1:**
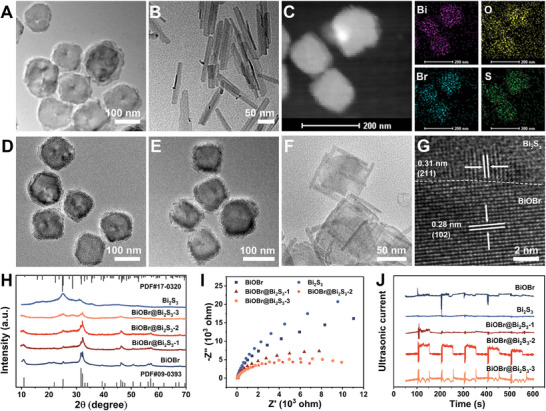
TEM images of A) BiOBr nanosheets and B) Bi_2_S_3_ nanorods. C) HAADF image of S‐scheme BiOBr@Bi_2_S_3_ nanoheterojunctions and the corresponding elemental mapping images. D–F) TEM images of BiOBr@Bi_2_S_3_‐1 (D), BiOBr@Bi_2_S_3_‐2 (E), and BiOBr@Bi_2_S_3_‐3 (F). G) HRTEM image of the BiOBr@Bi_2_S_3_‐2. H–J) XRD analysis (H), EIS spectra (I), and transient ultrasonic current spectra (J) of BiOBr, Bi_2_S_3_, BiOBr@Bi_2_S_3_‐1, BiOBr@Bi_2_S_3_‐2, and BiOBr@Bi_2_S_3_‐3.

BiOBr nanosheets and Bi_2_S_3_ nanorods were synthesized using a typical hot injection preparation process. Transmission electron microscopy (TEM) images reveal that the mean particle size of the BiOBr nanosheets is ≈115 nm (**Figure** **1**A), and the length of the Bi_2_S_3_ nanorods is ≈100 nm (Figure 1B). High‐angle annular dark‐field (HAADF) images and corresponding elemental mapping images suggest that elements are evenly distributed (Figures S1 and S2, Supporting Information). In the high‐resolution transmission electron microscope (HRTEM) image, the crystal face spacing of BiOBr is 0.28 nm, corresponding to (102) crystal faces (Figure S3A, Supporting Information). Meanwhile, the HRTEM image of Bi_2_S_3_ nanorods displays that the crystal face spacing is 0.31 nm corresponding to (211) crystal faces (Figure S3B, Supporting Information). Subsequently, BiOBr@Bi_2_S_3_ nanoheterojunctions were generated in situ by a one‐step anion exchange reaction based on the synthesis of BiOBr nanosheets. In order to find the optimal ratio of BiOBr nanosheets to Bi_2_S_3_ nanorods in BiOBr@Bi_2_S_3_ nanoheterojunction, we constructed three kinds of heterostructures by adjusting the amount of thioacetamide (TAA) added (0.25, 0.5, and 0.75 mmol), which were named BiOBr@Bi_2_S_3_‐1 (Figure 1D), BiOBr@Bi_2_S_3_‐2 (Figure 1E), and BiOBr@Bi_2_S_3_‐3 (Figure 1F), respectively. The TEM images clearly verify the formation of nanoheterojunction. As displayed in Figure 1D–F, Bi_2_S_3_ nanorods are successfully grown on the edge of the BiOBr nanosheet, and the length increases with the increasing amount of TAA. Moreover, the average particle size of these nanoheterojunctions was ≈110, 100, and 80 nm, respectively. The HRTEM images indicate d‐spacing of 0.28 and 0.31 nm, corresponding to the planes of BiOBr nanosheets and Bi_2_S_3_ nanorods, respectively (Figure 1G), and HAADF images and corresponding elemental mapping images observed in Figure 1C exhibit that Bi, O, Br, and S are evenly distributed in the BiOBr@Bi_2_S_3_ nanoheterojunctions. At the same time, the ratios of Bi, O, Br, and S in the nanoheterojunctions were characterized by energy dispersive spectroscopy (EDS), manifesting that the ratios of BiOBr and Bi_2_S_3_ in the three heterojunctions were 5:1, 2:1 and 1:2, respectively (Figure S4, Supporting Information). Then, the crystal phase structure of the nanomaterials was characterized by X‐ray powder diffraction (XRD). As provided in Figure 1H, the diffraction peaks of BiOBr and Bi_2_S_3_ are matched with the characteristic peaks of standard BiOBr (JCPDS: 09–0393) and Bi_2_S_3_ (JCPDS: 17–0320), respectively. Importantly, the diffraction peaks of BiOBr@Bi_2_S_3_ nanoheterojunction were composed of the characteristic diffraction peaks of BiOBr and Bi_2_S_3_, and the intensity of the diffraction peaks of Bi_2_S_3_ gradually increased with the addition of TAA. Besides, the position of diffraction peaks of nanoheterojunction did not change with the growth of Bi_2_S_3_ nanorods, indicating that the crystal structure did not change with the formation of heterojunction. All the above characterization can prove the successful construction of nanoheterojunctions. In order to sift the heterostructures with the optimal performance, electrochemical characterization was carried out. In electrochemical impedance spectroscopy (EIS), the semicircle of BiOBr@Bi_2_S_3_ nanoheterojunctions is smaller than that of BiOBr nanosheets and Bi_2_S_3_ nanorods, and the semicircle of BiOBr@Bi_2_S_3_‐2 nanoheterojunctions is the smallest, meaning that the sonoexcited electrons can be transmitted with the least obstruction and the fastest speed (Figure 1I). In addition, the transient ultrasonic current response study demonstrated that the ultrasonic current intensity of nanoheterojunctions was stronger (Figure 1J). Moreover, when the ratio of BiOBr nanosheets to Bi_2_S_3_ nanorods was 2 to 1, the ultrasonic current intensity of nanoheterojunctions was the highest, proving that BiOBr@Bi_2_S_3_‐2 nanoheterojunctions could generate more excited electrons under the same US irradiation condition. To further verify this conclusion, we also tested the photocurrent response of the samples, revealing that BiOBr@Bi_2_S_3_‐2 had the largest photocurrent density and the highest carrier separation efficiency (Figure S5, Supporting Information). These results strongly prove that the formation of BiOBr@Bi_2_S_3_‐2 nanoheterojunctions can effectively avoid electron‐hole recombination and promote carrier transfer generated by the US. Therefore, in the follow‐up experiment, we chose BiOBr@Bi_2_S_3_‐2 nanoheterojunctions to study anticancer effect and called them BiOBr@Bi_2_S_3_ nanoheterojunction for short. To enhance the hydrophilicity and biocompatibility, the surface of BiOBr@Bi_2_S_3_ nanoheterojunctions was functionalized by DSPE‐PEG_2000_. The characteristic absorption peaks of DSPE‐PEG_2000_, O‐CH_2_ (1109 cm^−1^), and NH‐C = O (1739 cm^−1^), can be observed from the Fourier transform infrared (FT‐IR) spectroscopy of DSPE‐PEG_2000_ and BiOBr@Bi_2_S_3_‐DSPE‐PEG_2000_, suggesting that DSPE‐PEG_2000_ is successfully modified on the surface of BiOBr@Bi_2_S_3_ nanoheterojunctions (Figure S6, Supporting Information). The Zeta potential analysis displayed that functionalized nanoparticles were negatively charged (Figure S7, Supporting Information). Meanwhile, the nanoparticles could maintain good stability in fetal bovine serum (Figure S8, Supporting Information). These results all indicate that DSPE‐PEG_2000_‐modified nanoparticles have potential for biological applications. Finally, we evaluated the degradability of BiOBr@Bi_2_S_3_ S‐scheme heterojunction in the biomicroenvironment. The results revealed that the nanoheterojunction was not biodegradable (Figure S9, Supporting Information).

**Figure 2:**
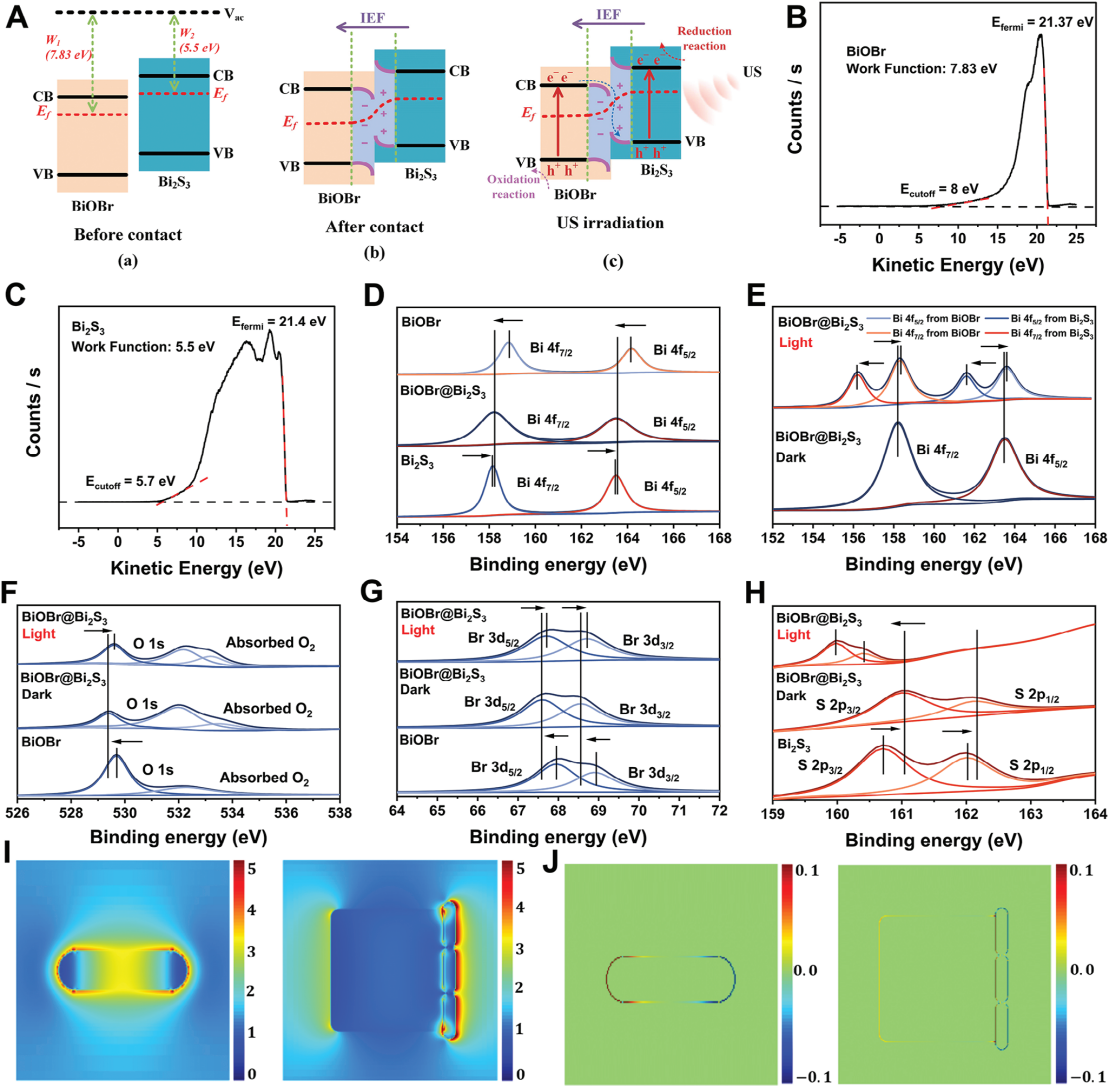
A) Schematic illustration of the S‐scheme transfer mechanism between BiOBr nanosheets and Bi_2_S_3_ nanorods. B,C) *W_f_* of BiOBr nanosheets (B) and Bi_2_S_3_ nanorods (C). D‐H) High‐resolution XPS spectra of Bi 4f of BiOBr, Bi_2_S_3_, and BiOBr@Bi_2_S_3_ D,E), O 1s (F) and Br 3d (G) of BiOBr and BiOBr@Bi_2_S_3_, and S 2p of Bi_2_S_3_ and BiOBr@Bi_2_S_3_ (H). I,J) The relative electric field intensities (I) and surface charge distribution (J) of the Bi_2_S_3_ nanorods and BiOBr@Bi_2_S_3_ heterojunction.

Next, the charge transfer mechanism between BiOBr nanosheets and Bi_2_S_3_ nanorods was investigated in depth. When two kinds of semiconductor nanocatalysts are in contact, the difference of *W* or *E_f_* determines the direction of charge transfer.^[^ ^22^ ^]^ Therefore, the direction of electron transfer within the heterojunction was first determined by the characterization of *W* and *E_f_*. The ultraviolet photoelectron spectroscopy (UPS) reveals that the *W* of BiOBr, Bi_2_S_3,_ and BiOBr@Bi_2_S_3_ are 7.83, 5.5, and 5.75 eV, respectively (**Figure** **2**B,C and Figure S10, Supporting Information). Simultaneously, the *E_f_* of BiOBr, Bi_2_S_3_, and BiOBr@Bi_2_S_3_ are 21.37, 21.4, and 21.3 eV, respectively. Obviously, the *W* of Bi_2_S_3_ is smaller than that of BiOBr, while its *E_f_* is larger than that of BiOBr. Therefore, when the BiOBr nanosheets combined with the Bi_2_S_3_ nanorods to form heterojunctions, electrons of Bi_2_S_3_ were easily transferred to BiOBr. Due to electron loss, the Bi_2_S_3_ side carried positive charges at the interface, resulting in an upward band bending, while the BiOBr side carried negative charges due to electron accumulation, causing a downward band bending (Figure 2A). In consequence, the outcome of charge transfer was the formation of an IEF from Bi_2_S_3_ to BiOBr at the interface between Bi_2_S_3_ and BiOBr. Concurrently, the gradient distribution of *E_f_* from the interface to the bulk region was established due to the spontaneous transfer of electrons. When BiOBr@Bi_2_S_3_ nanoheterojunctions were exposed to US irradiation, electrons of BiOBr nanosheets and Bi_2_S_3_ nanorods were excited, respectively. Under the combined action of IEF and interfacial band bending, the sonoexcited electrons on the CB of BiOBr easily recombined with the sonoexcited holes on the VB of Bi_2_S_3_ at the interface, while the holes on VB of BiOBr and the electrons on CB of Bi_2_S_3_ were retained to participate in a redox reaction, respectively. Note that this mode of charge transfer behavior conforms to the S‐scheme mechanism rather than Z‐scheme. To further substantiate this electron transfer mechanism, the XPS was employed to analyze the surface elements and chemical states of BiOBr, Bi_2_S_3_, and BiOBr@Bi_2_S_3_. This technique is particularly valuable as the binding energy of inner electrons in an element is influenced by the shielding effect of outer electrons and the attractive forces of the nucleus. Consequently, changes in the binding energy directly reflect alterations in electron density. In other words, variations in the density of the outer electron cloud result in corresponding increases or decreases in the binding energy of inner electrons. The Bi 4f high‐resolution XPS analysis showed that the characteristic peaks of Bi 4*f_7/2_* and Bi 4*f_5/2_* in BiOBr nanosheets were located at 158.8 and 164.2 eV, respectively and the characteristic peaks of Bi in Bi_2_S_3_ nanorods were 158.2 and 163.5 eV, respectively (Figure 2D). When BiOBr combined with Bi_2_S_3_ to form heterojunction, the XPS spectra of Bi 4f for BiOBr@Bi_2_S_3_ were located at 158.3 and 163.6 eV, respectively. Notably, these characteristic peaks of Bi 4f shifted toward lower binding energy compared to the original BiOBr, while shifted toward higher binding energy compared to the original Bi_2_S_3_. Similarly, compared with the O 1s (529.7 eV) and Br 3d (3*d_5/2_*: 68 eV, 3*d_3/2_*: 68.9 eV) characteristic peaks in BiOBr, these characteristic peaks in BiOBr@Bi_2_S_3_ (O 1s: 529.4 eV, Br 3*d_5/2_*: 67.6 eV, 3*d_3/2_*: 68.6 eV) all moved toward the direction of low binding energy (Figure 2F,G). However, compared with Bi_2_S_3_ (S 2*p_3/2_*: 160.7 eV, 2*p_1/2_*: 162 eV), the S 2p characteristic peaks in BiOBr@Bi_2_S_3_ (2*p_3/2_*: 161 eV, 2*p_1/2_*: 162.2 eV) all moved toward the direction of high binding energy (Figure 2H). These results demonstrate that when BiOBr is combined with Bi_2_S_3_, the electron density of BiOBr increase while the electron cloud density of Bi_2_S_3_ decreases, which means that electrons are transferred from Bi_2_S_3_ to BiOBr. Furthermore, in order to verify the electron transfer during US irradiation, UV–Vis light was used instead of US for in situ irradiated XPS measurement. Compared with BiOBr@Bi_2_S_3_ in darkness, the binding energies of Bi 4f (ascribed to BiOBr, Bi 4*f_7/2_*: 158.4 eV, 4*f_5/2_*: 163.7 eV), O 1s (529.6 eV), and Br 3d (3*d_5/2_*: 67.7 eV, 3*d_3/2_*: 68.7 eV) in BiOBr@Bi_2_S_3_ nanoheterojunctions under light irradiation moved significantly to higher energy levels (Figure 2E,G), while the binding energies of Bi 4f (ascribed to Bi_2_S_3_, Bi 4*f_7/2_*: 151.2 eV, 4*f_5/2_*: 161.6 eV) and S 2p (S 2*p_3/2_*:160 eV, 2*p_1/2_*:160.4 eV) moved significantly to lower energy levels (Figure 2E,H), indicating the transfer of photogenerated electrons from BiOBr to Bi_2_S_3_. These XPS data reveal a charges transfer pathway at the interface between BiOBr and Bi_2_S_3_, providing direct evidence for the successful construction of S‐scheme BiOBr@Bi_2_S_3_ heterojunctions.

In order to further investigate the surface charge distribution under external stimulation, the relative electric field intensity and surface charge in the Bi_2_S_3_ nanorods and BiOBr@Bi_2_S_3_ S‐scheme heterojunctions were estimated by the finite element method. As shown in Figure 2I, the electric field in the Bi_2_S_3_ nanorods is symmetrically distributed. However, the electric field distribution is asymmetrical after the formation of heterojunction, and has higher intensity at the interface and surface. This means the dramatic migration of electrons toward Bi_2_S_3_ nanorods. The results of charge distribution show that a large number of positive and negative charges accumulate at the heterojunction interface, while the surface of Bi_2_S_3_ is mainly negative, and the surface of BiOBr is mainly positive (Figure 2J). These data confirm that the construction of heterojunction is conducive to excited charge separation and provides theoretical support for the S‐scheme charge transfer mechanism. Therefore, our work provides a paradigm for the construction and characterization of S‐scheme nanoheterojunctions.

**Figure 3:**
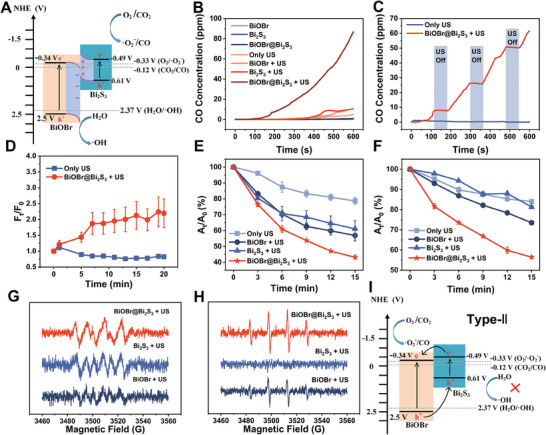
A) Band structures of S‐scheme heterojunction. B,C) Sonocatalytic CO generation behavior of the BiOBr, Bi_2_S_3_, and BiOBr@Bi_2_S_3_ nanocatalyst with or without US irradiation. D) The detection of CO generation by FL‐CO‐1 probe (mean ± S.D., *n* = 3). E) Comparison of SDT performance of BiOBr, Bi_2_S_3_, and BiOBr@Bi_2_S_3_ using DPBF probe (mean ± S.D., *n* = 3). F) Determination of •OH generation through degradation of MB (mean ± S.D., *n* = 3). G,H) Comparison of •O_2_^−^ (G) and •OH (H) generation for BiOBr, Bi_2_S_3_, and BiOBr@Bi_2_S_3_ under US irradiation demonstrated by ESR spectra. I) Schematic diagrams of conventional type‐II heterojunction.

Compared with common type‐II and Z‐scheme heterojunctions, S‐scheme heterojunctions can retain the holes and electrons with strong redox capacity, while the weak carriers are recombined, which helps to broaden the range of catalytic products. To investigate the sonocatalytic products of BiOBr@Bi_2_S_3_ S‐scheme heterojunction, the CB and VB positions, as well as the bandgaps (*E_g_*), were determined through Mott–Schottky (M–S) and UV–Vis diffuse reflectance spectra (UV‐Vis DRS) characterizations. The M–S curves of BiOBr and Bi_2_S_3_ both had positive slopes, which was a typical characteristic of n‐type semiconductors (Figure S11A,B, Supporting Information). Moreover, the flat‐band potentials (*E_fb_*) of BOBr and Bi_2_S_3_ could be calculated as −0.75 and −0.9 V (vs normal hydrogen electrode (NHE), pH 7), respectively, through the intersection of the tangent line and the coordinate axis. In general, the CB potential of n‐type semiconductor is close to the *E_fb_*. Consequently, the CB potentials of BiOBr and Bi_2_S_3_ were −0.75 and −0.9 V (vs NHE, pH 7), respectively, and were converted to −0.34 and −0.49 V (vs NHE, pH 0) according to Nernst equation (The potentials used below are all relative to NHE pH 0.). Besides, the *E_g_* of BiOBr and Bi_2_S_3_ were 2.84 and 1.7 eV, respectively, as obtained by UV–vis DRS characterization (Figure S11C,D, Supporting Information). Hence, the VB potentials of BiOBr and Bi_2_S_3_ were computed to be 2.5 and 0.61 V, respectively. According to the above‐mentioned conclusion, the band structures of BiOBr and Bi_2_S_3_ are depicted in **Figure** **3**A. From the scheme of the band structure, it can be clearly seen that the CB edge of Bi_2_S_3_ is more negative than the redox potentials of O_2_/•O_2_^−^ (−0.33 V) and CO_2_/CO (−0.12 V), while the VB edge of BiOBr is more positive than the redox potential of H_2_O/•OH (2.37 V). This indicates that it is thermodynamically feasible for BiOBr@Bi_2_S_3_ S‐scheme heterojunctions to catalyze the generation of CO and ROS under US irradiation.

In short, when BiOBr combines with Bi_2_S_3_ to form S‐scheme heterojunction, the electrons of Bi_2_S_3_ will transfer to BiOBr, and IEF and band bending at the interface are formed. When the S‐scheme heterojunctions are irradiated by US, the electrons of BiOBr and Bi_2_S_3_ are excited and transition to CB. Driven by IEF and band bending, the electrons of BiOBr recombine with the holes of Bi_2_S_3_, while the holes of BiOBr and electrons of Bi_2_S_3_ are retained, catalyzing the formation of •OH, CO, and •O_2_^−^, respectively. It can be seen that this unique S‐scheme electron transport pathway improves charge utilization and thus achieves efficient sonocatalytic therapy. To further verify the high efficiency of the S‐scheme BiOBr@Bi_2_S_3_ nanoheterojunctions, the sonocatalytic performance was investigated. A commercial portable CO detector was used to detection the CO generation of the S‐scheme BiOBr@Bi_2_S_3_ nanoheterojunctions under US irradiation. From Figure 3B, US alone, BiOBr, Bi_2_S_3_, BiOBr@Bi_2_S_3_, BiOBr + US, and Bi_2_S_3_ + US cannot detect the sufficient amount of CO, while BiOBr@Bi_2_S_3_ nanoheterojunction exhibit efficient CO generation under US irradiation. It was important that the CO generation was highly controllable and repeatable by turning on/off the US therapeutic instrument, which indicated the way of sonocatalytic CO_2_ reduction could enable on‐demand CO therapy (Figure 3C). Meanwhile, the fluorescence intensity of the CO fluorescent probe, allyl chloroformate functionalized fluorescein (FL‐CO‐1), continued to enhance as US irradiation progresses, which also indicated that BiOBr@Bi_2_S_3_ had the ability to produce CO under US irradiation (Figure 3D and (Figure S12, Supporting Information). Next, the diphenyl isobenzofuran (DPBF) probe was applied to detect ROS generation. Similar to the case of CO generation, ROS generation was detected in all experimental groups, among which the BiOBr@Bi_2_S_3_ nanoheterojunctions coupling US group had stronger ROS generation ability than other groups (Figure 3E; Figure S13, Supporting Information). Subsequently, the •O_2_^−^ generation in each group was detected by electron spin resonance (ESR) using 5, 5‐dimethyl‐1‐pyrroline N‐oxide (DMPO) as the trapping agent. The •O_2_^−^ signal peaks in BiOBr@Bi_2_S_3_ + US irradiation group were the strongest, compared with the BiOBr, Bi_2_S_3_, BiOBr@Bi_2_S_3_, BiOBr + US, and Bi_2_S_3_ + US groups (Figure 3G; Figure S15A, Supporting Information). It was evident that the sonoexcited electrons retained in the S‐scheme heterojunction could effectively reduce CO_2_ and O_2_. Numerous studies have confirmed that the •O_2_^−^ production efficiency was constrained by the concentration of O_2_ in the surrounding environment.^[^ ^23^ ^]^ This implies that even with the optimization of sonocatalyst, the generation of •O_2_^−^ will be restricted due to the nature of the hypoxic tumor microenvironment, consequently leading to a suboptimal therapeutic effect. Therefore, it is not advisable to solely focus on enhancing the performance of sonocatalyst, while ignore the influence of the tumor microenvironment. Fortunately, from the perspective of reaction substrate, CO_2_ reduction is not limited by O_2_ concentration. Therefore, the strategy of concurrent CO and •O_2_^−^ generation can compromise the disadvantage of insufficient •O_2_^−^ generation in hypoxic environment. Moreover, methylene blue (MB) was chosen to characterize the ability of BiOBr nanosheets, Bi_2_S_3_ nanorods, and BiOBr@Bi_2_S_3_ nanoheterojunctions to generate •OH under US irradiation. The dates suggested that BiOBr@Bi_2_S_3_ nanoheterojunctions had the highest •OH production (Figure 3F; Figure S14, Supporting Information). In addition, the presence of a typical ESR signal with an intensity ratio of 1:2:2:1 also indicated that the ability of BiOBr@Bi_2_S_3_ to generate •OH, and the significantly higher signal peak intensity compared with BiOBr and Bi_2_S_3_ proved the prominent sonocatalytic performance of S‐scheme heterojunctions (Figure 3H; Figure S15B, Supporting Information). It was noteworthy that the signal peak corresponding to •OH was hardly detected in Bi_2_S_3_ + US group, which was expected in theory given its more negative VB edge position (0.61 V) compared to the redox potential of •OH (2.37 V). This observation further implied that the charge transfer mechanism of the BiOBr@Bi_2_S_3_ did not comply with that of the traditional type II heterojunction (Figure 3I). Otherwise, the system would be unable to generate •OH. From the perspective of catalytic products, it can also be inferred that the charge transfer pathway induced by US is more consistent with S‐scheme rather than type‐II.

**Figure 4:**
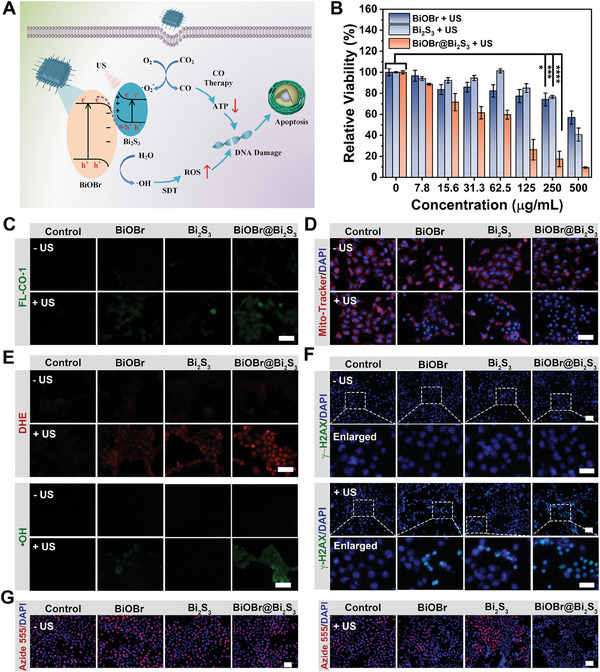
A) The proposed mechanism for combined CO therapy/SDT based on the BiOBr@Bi_2_S_3_ nanocatalyst. B) Cell viabilities of 4T1 cells after treated BiOBr, Bi_2_S_3_, and BiOBr@Bi_2_S_3_ with US irradiation (1.0 MHz, 1.0 W cm^−2^, 5 min) (mean ± S.D., *n* = 5). C–H) CO generation (C), mitochondrial damage (D), •O_2_^−^ and •OH generation (E), and early DNA damage (F) of 4T1 cells under various treatments. G) Fluorescence microscopy images of the reproductive capacity of 4T1 cells after various treatments. Note: The surfaces of BiOBr, Bi_2_S_3,_ and BiOBr@BiOBr used in the cell experiments were all modified with DSPE‐PEG_2000_. All scale bars are 25 µm. The *p* values were calculated by one‐way analysis of variance (ANOVA), ^****^ *p* < 0.0001, ^***^ *p* < 0.001, ^**^ *p* < 0.01, and ^*^ *p* < 0.05.

According to the above conclusions, the structure of S‐scheme heterojunctions facilitated the generation and separation of electrons and holes triggered by US, thus showing excellent sonocatalytic performance. Therefore, we continued to examine the in vitro catalytic properties and curative effects of nanoheterojunctions. The properties of cellular uptake of nanocatalysts were investigated first. Rhodamine B (RhB)–labeled nanoparticles were prepared and incubated with 4T1 mouse breast cancer cells to assess their uptake efficiency. As the incubation time increased, a more pronounced red fluorescence was observed, indicating the nanoparticles had time‐dependent cell uptake behavior (Figures S16–18, Supporting Information). Next, the biocompatibility of the nanoparticles was scored by Cell Counting Kit‐8 (CCK‐8) assay. As characterized in Figure S19,(Supporting Information), BiOBr nanosheets, Bi_2_S_3_ nanoorods, and BiOBr@Bi_2_S_3_ nanoheterojunctions have only slight effects on cell activity of normal L929 fibroblasts and 4T1 cells even at high concentrations, indicating high cytocompatibility and safety of nanocatalysts. In addition, US irradiation alone did not have a large effect on cell viability (Figure S20, Supporting Information). Subsequently, we evaluated the specific cytotoxicity of the nanocatalysts to cancer cells under US irradiation. BiOBr nanosheets, Bi_2_S_3_ nanoorods, and BiOBr@Bi_2_S_3_ nanoheterojunctions all exhibited concentration‐dependent cytotoxicity (**Figure** **4**B). But by contrast, BiOBr@Bi_2_S_3_ nanoheterojunctions showed the strongest cell‐killing effect. More than 83% of cancer cells died after being treated with BiOBr@Bi_2_S_3_ nanoheterojunction (250 µg mL^−1^) and US irradiation for 5 min. The flow cytometry technique was used to observe the cytotoxicity and apoptosis of cancer cells under different treatments, which were consistent with the cell viability data (Figure S21, Supporting Information). The main reason for the excellent anticancer properties of BiOBr@Bi_2_S_3_ nanoheterojunctions can be ascribed to the broadened range of catalytic substrates and product pools. This includes two reductions (O_2_/•O_2_^−^ and CO_2_/CO) and one oxidation (H_2_O/•OH) reaction.

To gain a deeper understanding of the mechanism of sonocatalytic therapy based on S‐scheme BiOBr@Bi_2_S_3_ nanoheterojunctions, we conducted further investigation into the effects of nanocatalysts on cancer cells under US irradiation. First, CO fluorescence probes (FL‐CO‐1) and ROS probes (2′,7′‐Dichlorofluorescein diacetate, DCFH‐DA) were employed to validate the CO_2_ reduction (Figure 4C) and ROS generation (Figure S22, Supporting Information) in 4T1 cells, which were responsible for the outcomes of sonocatalytic therapy. Furthermore, dihydroethidium (DHE) probes and •OH probes were used to monitor intracellular •O_2_^−^ and •OH levels, respectively. Figure 4E illustrates that •O_2_^−^ cannot be generated by US alone, or BiOBr nanosheets, Bi_2_S_3_ nanorods and BiOBr@Bi_2_S_3_ nanoheterojunctions in the absence of US treatments. However, •O_2_^−^ generation was detectable when nanocatalysts were coupled with US irradiation, in which BiOBr@Bi_2_S_3_ nanoheterojunctions demonstrated the strongest conversion ability. Similar results were obtained for the evaluation of intracellular •OH level. The BiOBr@Bi_2_S_3_ + US group exhibited the enhanced •OH generation outcome. The substantial increase in intracellular CO and ROS levels will lead to irreversible damage to mitochondrial and DNA. Therefore, the MitoTracker Deep Red, a mitochondrial fluorescence probe, along with an immunofluorescent staining kit of Phospho‐Histone H2AX (γ‐H2AX), a biomarker of double‐strand DNA breaks, were used to characterize mitochondrial and DNA damage. As depicted in Figure 4D and Figure S23 (Supporting Information), the quenching of red fluorescence from mitochondrial probes indicates that sonocatalytically generated CO seriously impairs the mitochondria in 4T1 cells, and thus causes a significant decrease in intracellular energy level (Figure S24, Supporting Information). On the other hand, the obvious green fluorescence appeared in the BiOBr@Bi_2_S_3_ + US group, indicating severe DNA damage, which can be attributed to the SDT effect (Figure 4F; Figure S25, Supporting Information). Importantly, the decline in intracellular energy levels mediated by CO not only accelerates the process of apoptosis but also prevents the repair of broken DNA, thereby synergistically enhancing the antitumor therapeutic effect of SDT.^[^ ^24^ ^]^ Besides, the consequences of nuclear staining proved that the nuclei of BiOBr@Bi_2_S_3_ + US group were significantly shrunk compared with other groups, which also strongly indicated that DNA was irreversibly damaged (Figure 4D,F,G; Figure S28, Supporting Information). At the same time, CO‐mediated intracellular energy decline can also inhibit the glycolysis process of cancer cells.^[^ ^25^ ^]^ As shown in Figures S26 and S27 (Supporting Information), the intracellular glucose relative amount in BiOBr@Bi_2_S_3_ + US group is significantly increased, while lactic acid content is decreased, indicating that glycolysis of tumor cells is obviously inhibited. It was worth mentioning that the reversal of glycolysis effectively inhibited the proliferation of cancer cells (Figure 4G; Figure S28, Supporting Information). By comparison, BiOBr nanosheets and Bi_2_S_3_ nanorods did not significantly improve intracellular levels of CO, ROS, and ATP under US irradiation, which was the main cause of poor therapeutic efficacy. The quantitative analysis of average fluorescence intensity provided direct evidence for the above conclusion (Figure S29, Supporting Information).^[^ ^26^ ^]^ Based on the above‐mentioned conclusion, we attributed the antitumor mechanism of BiOBr@Bi_2_S_3_ sonocatalyst to SDT/CO combination therapy (Figure 4A).

Subsequently, in vivo, therapeutic performances of the S‐scheme BiOBr@Bi_2_S_3_ nanoheterojunctions were investigated using a tumor mouse model. First, the inductively coupled plasma‐mass spectrometry (ICP‐MS) detection showed that BiOBr@Bi_2_S_3_ nanocatalyst could effectively accumulate at the tumor site in a passive targeting manner after intravenous injection, and the accumulation reached the maximum amount ≈24 h of the administration (Figure S30, Supporting Information). The determination of Bi ion content in feces showed that the injected nanoparticles were metabolized mainly through hepatobiliary elimination (Figure S31, Supporting Information). Besides, it can be seen from Figure S30 (Supporting Information) that BiOBr@Bi_2_S_3_ nanocomposites can also be distributed in other organs. Therefore, the biosafety of BiOBr@Bi_2_S_3_ nanocomposites was evaluated, which had important implications for subsequent in vivo therapy and clinical translation. The hematoxylin and eosin (H&E) section analysis was implemented on the major organs of mice at 1, 3, 5, 7, and 14 days after administration (Figure S32, Supporting Information). The outcomes clearly exhibited that there were no significant lesions in the vital organs such as the heart, liver, spleen, lung, and kidney, indicating that the BiOBr@Bi_2_S_3_ nanocomposites had good histocompatibility. At the same time, the test results of the general blood indexes (Figures S33 and S34, Supporting Information) proved that the BiOBr@Bi_2_S_3_ nanocomposite had good blood compatibility and not affect liver and kidney function.

**Figure 5:**
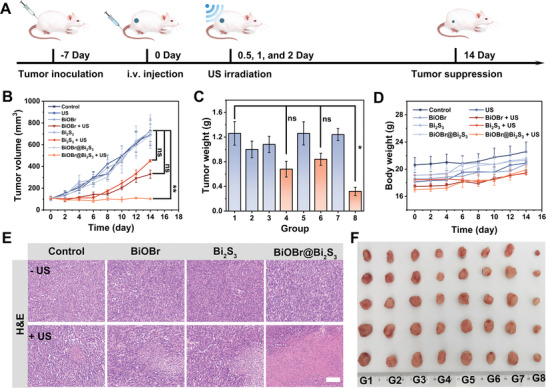
A) Therapeutic procedure for 4T1‐tumor‐bearing mice. B) Tumor volumes (mean ± SD, *n* = 5) of the mice in various treatment groups. C) Average tumor weight at the end of different treatments (mean ± SD, *n* = 5). D) Body weights (mean ± SD, *n* = 5) of the mice in various treatment groups. E) The H&E (scale bars = 100 µm) staining images of the tumor after different treatment groups. F) Photograph of tumors in different treatment groups. Note: The surfaces of BiOBr, Bi_2_S_3,_ and BiOBr@BiOBr were all modified with DSPE‐PEG_2000_. The *p* values were calculated by one‐way ANOVA, ^****^ *p* < 0.0001, ^***^ *p* < 0.001, ^**^ *p* < 0.01, and ^*^ *p* < 0.05. Group: 1) Control, 2) US, 3) BiOBr, 4) BiOBr + US, 5) Bi_2_S_3_, 6) Bi_2_S_3_ + US, 7) BiOBr@Bi_2_S_3_, 8) BiOBr@Bi_2_S_3_ + US.

Owing to the exceptional tumor accumulation property and biosafety of BiOBr@Bi_2_S_3_ nanocomposites, the antitumor effect of nanoparticles was further investigated. The tumor‐bearing mice were separated eight groups (*n* = 5) and given different treatments: 1) Control, 2) US, 3) BiOBr, 4) BiOBr + US, 5) Bi_2_S_3_, 6) Bi_2_S_3_ + US, 7) BiOBr@Bi_2_S_3_, and 8) BiOBr@Bi_2_S_3_ + US (**Figure** **5**A). The mice in groups 3, 4, 5, 6, 7, and 8 were injected intravenously with 10 mg Kg^−1^ nanoparticles. The US therapy in groups 4, 6, and 8 was accomplished at 12, 24, and 48 h after injection of nanoparticles. No significant restraint of tumor growth was observed in US only, BiOBr, Bi_2_S_3_, and BiOBr@Bi_2_S_3_ treated mice compared with the control group (Figure 5B). BiOBr and Bi_2_S_3_ were able to inhibit tumor growth to a certain extent when coupled with US irradiation, while BiOBr@Bi_2_S_3_ + US group demonstrated extreme inhabitation of tumor growth. Photographs of tumor‐bearing mice taken at the end of treatment (Figure S35, Supporting Information), as well as images of dissected tumors (Figure 5F), and measurements of tumor weight (Figure 5C), also provided direct evidence of the therapeutic outcomes of sonocatalytic therapy. Meanwhile, H&E staining analysis was performed to confirm the therapeutic effect of the respective treatment groups. From Figure 5E, the tumor cells in groups 4 and 6 show partial nuclear pyknosis or no nucleus, while the majority of tumor cells in group 8 exhibit no nucleus. In contrast, no nuclear damage was observed in the other groups. In addition, the body weight of mice in each group had an upward tendency during the treatment period and no significant damage was observed in the major organs of the mice, indicating that these different treatments did not cause side effects (Figure 5D; Figure S36, Supporting Information). These outcomes collectively confirm that the rationally designed S‐scheme heterojunction can effectively inhibit tumor growth while ensuring safety within the tested range.

**Figure 6:**
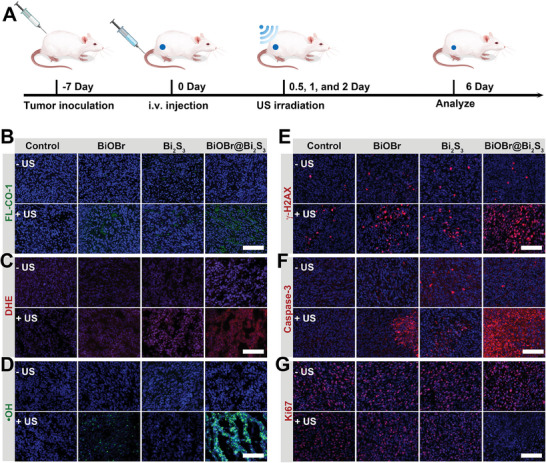
A) Assessment protocol of antitumor mechanism in vivo. B–G) Immunofluorescence images of the generated CO were stained with FL‐CO‐1 (B), the generated •O_2_^−^ was stained with DHE (C), the generated •OH was stained with •OH probe (D), the damaged DNA was stained with γ‐H2AX (E), the apoptotic cells were stained using the apoptosis marker Caspase‐3 (F), proliferating cells was stained with Ki67 (G). Note: The surfaces of BiOBr, Bi_2_S_3,_ and BiOBr@BiOBr were all modified with DSPE‐PEG_2000_. All scale bars are 50 µm.

To further elucidate the mechanism proposed‐above for combined SDT/CO therapy, immunofluorescence staining was used to analyze the levels of CO and ROS within the tumor, as well as assess DNA damage and cell apoptosis (**Figure** **6**A). In the DHE and •OH staining analysis, •O_2_^−^ signal was observed in both groups 4 and 6, while •OH signal was only observed in group 4 (Figure 6C,D; Figure S38, Supporting Information). However, enhanced •O_2_^−^ and •OH signals were significantly observed in group 8, which were attributed to effective SDT performance. Correspondingly, after 1.0 W cm^−2^ US irradiation on tumors for 10 min, CO generation could be detected in groups 4, 6, and 8, and the ability of CO generation was strongest in group 8, indicating that the BiOBr@Bi_2_S_3_ nanoheterojunctions possessed high efficiency in generating sonocatalytic CO intratumorally (Figure 6B and Figure S38, Supporting Information). Meanwhile, the great increase of intratumoral ROS level intensively (Figure S37, Supporting Information) led to DNA damage and thus induced tumor apoptosis (Figure 6E; Figure S39, Supporting Information), while sonocatalytically generated CO cut off the energy supply of DNA repair (Figure S40, Supporting Information) and thus accelerated the apoptosis process (Figure 6F; Figure S39, Supporting Information). Under the mechanism of combined SDT/CO therapy, tumor proliferation was effectively suppressed and demonstrated through Ki67 cellular staining (Figure 6G; Figure S39, Supporting Information). All of these in vivo analyses collectively suggest that the S‐scheme BiOBr@Bi_2_S_3_ nanoheterojunctions exhibited excellent sonocatalytic properties under US irradiation and can mediate effective cancer therapy.

## 3 Conclusion

At present, there are two main problems in heterojunction catalytic therapy: 1) both conventional type‐II and Z‐scheme charge transfer mechanisms have the potential to reduce the redox capacity of nanomaterials; 2) the types of catalytic substrates in the tumor microenvironment are minimal, resulting in a single treatment mode and limited outcomes. Therefore, in this study, a S‐scheme BiOBr@Bi_2_S_3_ nanoheterojunction was developed to address these two problems, which could promote charge separation and broaden the selectivity of catalytic substrates. In this nanoheterojunctions, BiOBr nanosheets and Bi_2_S_3_ nanorods with different *E_f_* and band structures were in contact with each other to induce charge redistribution at the interface, thus mediating IEF construction. Under US irradiation, the sonoexcited electrons of BiOBr recombined with sonoexcited holes on the VB of Bi_2_S_3_ under the guidance of IEF, leaving holes and electrons with strong redox capacity on the VB of BiOBr and the CB of Bi_2_S_3_, respectively. Meanwhile, the band bending and IEF also effectively blocked the sonoexcited electrons flow from Bi_2_S_3_ to BiOBr. Therefore, the design of S‐scheme heterostructure can enhance the charge utilization while preserving the redox capacity of the catalyst. Importantly, the retained high‐energy electrons and holes had the ability to catalyze various substrates, including the reduction of O_2_ and CO_2_ to •O_2_^−^ and CO, and the oxidation of H_2_O to •OH. Both in vitro and in vivo, BiOBr@Bi_2_S_3_ nanocatalysts demonstrated the ability to control the generation of ROS and CO under US irradiation. This protocol effectively inhibited tumor growth and proliferation by inducing DNA damage and depressing cancer cell energy metabolism, thereby achieving the desired goal of SDT/CO combined therapy. In conclusion, this work provides an alternative strategy to solve the plaguing issue of the limited efficiency of sonocatalytic therapy from a material design perspective, with the aim of advancing the development of nanocatalytic medicine.

## Conflict of Interest

The authors declare no conflict of interest.

## Supporting information

Supporting Information

## Data Availability

The data that support the findings of this study are available from the corresponding author upon reasonable request.
