# Supplementary material for: Fabrication of Interface Engineered S‐Scheme Heterojunction Nanocatalyst for Ultrasound‐Triggered Sustainable Cancer Therapy
Source: Adv Sci (Weinh). 2024 Feb 11;11(15):2308546. doi: 10.1002/advs.202308546 (PMC11022741; doi:10.1002/advs.202308546)
Supplement: Supplementary file 1 — Supporting Information [file ADVS-11-2308546-s001.pdf]

ADVANCED SCIENCE
Open Access

Supporting Information

for *Adv. Sci.*, DOI 10.1002/advs.202308546

Fabrication of Interface Engineered S-Scheme Heterojunction Nanocatalyst for Ultrasound-Triggered Sustainable Cancer Therapy

Meng Yuan, Ling Yang, Zhuang Yang, Zhizi Ma, Jie Ma, Zhendong Liu, Ping'an Ma, Ziyong Cheng*, Aziz Maleki and Jun Lin**

Supporting Information

Fabrication of Interface Engineered S-scheme Heterojunction Nanocatalyst for Ultrasound-Triggered Sustainable Cancer Therapy

Meng Yuan, Ling Yang, Zhuang Yang, Zhizi Ma, Jie Ma, Zhendong Liu, Ping'an Ma, Ziyong Cheng,* Aziz Maleki, and Jun Lin**

Dr. M. Yuan, Dr. L. Yang, Dr. Z. Yang, Dr. Z. Ma, Dr. J. Ma, Dr. Z. Liu, Prof. P. Ma, and Prof. J. Lin.
State Key Laboratory of Rare Earth Resource Utilization, Changchun Institute of Applied Chemistry, Chinese Academy of Sciences, Changchun 130022, China
E-mail: mapa675@ciac.ac.cn; jlin@ciac.ac.cn

Dr. M. Yuan, Dr. L. Yang, Dr. Z. Yang, Dr. Z. Ma, Dr. J. Ma, Dr. Z. Liu, Prof. P. Ma, and Prof. J. Lin.
School of Applied Chemistry and Engineering, University of Science and Technology of China, Hefei 230026, China

Prof. Z. Cheng
Key Laboratory of Superlight Materials and Surface Technology, Ministry of Education, College of Materials Science and Chemical Engineering, Harbin Engineering University, Harbin 150001, China
E-mail: zycheng@ciac.ac.cn

Dr. A. Maleki
Zanjan Pharmaceutical Nanotechnology Research Center (ZPNRC), and Department of Pharmaceutical Nanotechnology (School of pharmacy), Zanjan University of Medical Sciences, Zanjan 4513956184, Iran

Experimental Section

Materials: $Bi(NO_3)_3 \cdot 5H_2O$, Thioacetamide (TAA), DSPE-PEG_{2000}, DSPE-PEG-NH_2 (M_w = 2000), and Rhodamine B (RhB) were purchased from Aladdin Industrial Corporation. Oleylamine and oleic acid were purchased from Sigma-Aldrich. Octadecene were purchased from Adamas-beta. Potassium bromide were purchased from Xilong Scientific Co., Ltd. All chemicals were used as received without further purification.

Measurements and characterizations: The transmission electron microscopy (TEM) and high-resolution transmission electron microscopy (HRTEM) of samples were imaged using a FEI Tecnai G2 S-Twin with a field emission gun operating at 200 kV. X-ray diffraction measurements (XRD) were performed on a D8 Advanced diffractometer (Bruker) using CuKa radiation (λ = 0.154 nm). The inductively coupled plasma-mass spectrometer (ICP-MS) was taken on an Icap 6300 of Thermo Scientific. X-ray photoelectron spectroscopy (XPS) spectra were obtained with thermo scientific K-Alpha using Al Kα (1486.6 eV) as the excitation source. The zeta potential and dynamic light scattering of nanoparticles in H_2O was obtained on a Zetasizer 3000HS analyzer. Electrochemical workstation is CHI660E. Chattanooga-2776 sonicator was used to generate ultrasound during the treatment.

Preparation of step-scheme (S-scheme) BiOBr@Bi_2S_3 heterojunction nanocomposites: the S-scheme BiOBr@Bi_2S_3 heterojunction was synthesized by a typical hot injection method. Added 0.5 mmol $Bi(NO_3)_3 \cdot 5H_2O$, 10 mL octadecene, 1 mL oleic acid, and 1 mL oleamine to a 100 mL three-neck bottle. The mixture was then heated to 175 °C in a nitrogen atmosphere and stirred. Stop heating until $Bi(NO_3)_3 \cdot 5H_2O$ was completely dissolved. When the temperature dropped to 130 °C, KBr aqueous solution (4 mmol KBr dissolved in 10 mL of deionized water) was injected quickly and reacted at 100 °C for 30 min. The TAA solution (1 M) was then slowly added at a rate of 25 μL/min. Finally, the heating was stopped and the product was cooled to room temperature. To purify the product, 10 mL ethanol was added to the product and mixed evenly. The mixture was allowed to stand for delamination before collecting the upper oil phase and discarding the lower water phase. Then, 10 mL of deionized water and 10 mL of ethanol were added to the upper oil phase. This step needs to be repeated 3 times. Finally, 2 mL ethanol was added to the oil phase solution and the mixture was centrifuged at 8000 rpm for 5 min. In order to synthesize heterojunction with different Bi_2S_3 content, we adjusted the amount of TAA. When the amounts of TAA were 0.25 mmol, 0.5 mmol, and 0.75 mmol, the products were named BiOBr@Bi_2S_3-1, BiOBr@Bi_2S_3-2, and BiOBr@Bi_2S_3-3, respectively. BiOBr nanosheets and Bi_2S_3 nanorods were synthesized by the same method without TAA or KBr.

Surface modification: Simply, 10 mg BiOBr nanosheets or Bi_2S_3 nanorods or BiOBr@Bi_2S_3 nanoheterojunctions were mixed with 15 mg DSPE-PEG_{2000} in 5mL chloroform and ultrasonically dispersed for 5 min. After stirring overnight, the solvent could be volatilized by gently stirring to obtain water-soluble nanoparticles.

Synthesis RhB modified nanoparticles: For the labeling of nanoparticles with RhB, DSPE-PEG-NH_2 was modified on the surface of BiOBr nanosheets, Bi_2S_3 nanorods and BiOBr@Bi_2S_3 nanoheterojunctions using the same procedure as above. Meanwhile, 5 mg RhB was added in 5 mL deionized water containing 10 mg 1-(3-Dimethylaminopropyl)-3-ethylcarbodiimide hydrochloride (EDC) and 15 mg N-Hydroxy succinimide (NHS) and stirred in the dark for 30 min. Afterward, DSPE-PEG-NH_2-modified nanoparticles were added to the mixed solution and stirred in the dark for 24 h. Finally, the products were collected by centrifugation and washed several times with deionized water.

Electric field and charge distribution simulation: We have calculated the electric field distribution and charge distribution of the nanostructures using the finite element method. During the calculations, we set both x, y and z directions as perfectly matched layer conditions to prevent unphysical scattering. In addition, we divided the whole simulation area into a 0.25 nm*0.25 nm*0.25 nm mesh to ensure accurate calculation results. Importantly, the total field scattering field light source is used as an excitation light source incident perpendicularly along the z-direction to the surface of the nanostructures. Finally, the electric field distribution and charge distribution of the nanostructures in the x-y plane were obtained using an electric field monitor and a charge monitor.

Detection of reactive oxygen species (ROS) generation in vitro: DPBF is a typical molecular probe for detecting ROS production. 6 μL DPBF (10 mg/mL) was added to 2 mL BiOBr, Bi_2S_3, or BiOBr@Bi_2S_3 solution (250 μg/mL). The mixture was then subjected to ultrasound (US) irradiation (1 MHz, 50% duty cycle, 1.0 W cm^{-2}) in a dark environment and the absorption of DPBF was recorded by UV-Vis spectroscopy very 3 min.

MB was applied as a hydroxyl radical (•OH) indicator for detecting the generation of BiOBr, Bi_2S_3, or BiOBr@Bi_2S_3. 6 μL MB (1 mg/mL) was added to 2 mL BiOBr, Bi_2S_3, or BiOBr@Bi_2S_3 solution (250 μg/mL). The mixture was then subjected to US irradiation (1 MHz, 50% duty cycle, 1.0 W cm^{-2}) in a dark environment and the absorption of MB was recorded by UV-Vis spectroscopy very 3 min.

Electron Spin Resonance (ESR) was used to further verifying the production of ROS by using a signal capture agent of 5,5-dimethyl-1-pyrroline-N-oxide (DMPO).

Detection of carbon monoxide (CO) production in vitro: Commercial portable CO

detectors are used to detect CO production. CO_2 was injected into BiOBr, Bi_2S_3, or BiOBr@Bi_2S_3 solution (250 μg/mL) by bubble method. After a certain amount of CO_2 was dissolved in the solution, the bubble was stopped and sealed, and the production of CO was measured with or without US irradiation (1 MHz, 50% duty cycle, 1.0 W/cm^2). Subsequently, we also used a CO fluorescent probe (allyl chloroformate functionalized fluorescein (FL-CO-1)) to verify that BiOBr@Bi_2S_3 could produce CO under US irradiation. FL-CO-1 and palladium chloride ($PdCl_2$) solution were added to CO_2-dissolved water or BiOBr@Bi_2S_3 solution (250 μg/mL) to achieve a final concentration of 5 μM, and the whole reaction system was sealed. The mixed solution was exposed to US irradiation (1 MHz, 50% duty cycle, 1.0 W cm^{-2}) and the fluorescence of FL-CO-1 was detected by fluorescence spectroscopy (λ_{ex} = 490 nm).

Electrochemical Measurements: Electrochemical measurements were carried out using an electrochemical workstation (CHI660E) in Na_2SO_4 electrolyte (0.5 M). The test system consisted of a working electrode (ITO), a reference electrode (Ag/AgCl), and an auxiliary plate (platinum plate). Subsequently, 10 mg powder sample was dispersed in 1 mL ethanol, and added 50 μL Nafion solution, ultrasonic for 30 minutes to form a uniform suspension, and then dropped 500 μL suspension on ITO glass and dried it at room temperature for testing. Subsequently, the electrolytic cell was placed in the ultrasonic cleaning machine (100 Hz) for US irradiation.

Evaluation of degradability of BiOBr@Bi_2S_3-DSPE-PEG_{2000}: BiOBr@Bi_2S_3-DSPE-PEG_{2000} (200 μg/mL) was dispersed in PBS, GSH solutions (5 mM), and acid buffers (pH = 6.5). The content of Bi^{3+} ion was detected by supernatant at a fixed time interval (0, 1, 3, 5, and 7 D).

Cell culture: L929 fibroblast cells were purchased from Procell Life Science & Technology Co., Ltd., 4T1 murine breast cancer cells were purchased from Shanghai Zhong qiao xin zhou Biotech and cultured in MEM and RPMI 1640 supplemented with 1% (v/v) penicillin/streptomycin and 10% (v/v) fetal bovine serum (FBS) at 37 °C under 5% CO_2. Trypsin-EDTA solution, 0.25% (without phenol red) were purchased from Beijing Solarbio Science & Technology Co., Ltd..

Cellular uptake behavior of nanoparticles: 4T1 cells was planted in 6-well plates and allowed to adhere for 12 h. After that, RhB-modified BiOBr, Bi_2S_3, and BiOBr@Bi_2S_3 (250 μg/mL) were added and co-incubated with cells for 0, 1, 2, and 4 h. Afterward, the cells were washed with PBS for three times and stained by Hoechst 33342 for 10 min before fluorescence images by fluorescence microscope.

In vitro cytotoxicity assay: Prior to verification the cytotoxicity, the biocompatibility of BiOBr, Bi_2S_3, and BiOBr@Bi_2S_3 were first evaluated using normal L929 cells and 4T1 cells by CCK-8. In brief, the cells were seeded in 96-well plates with the density of 8000 cells per well and grown in 5% CO_2 at 37 °C overnight. Then, different concentrations of nanoparticles were added to the medium, and the cells were incubated in 5% CO_2 at 37 °C for another 24 h. At the end of incubation, 10 μL of CCK-8 solution was added to each well and the cells were incubated for another 1 h. Finally, the plate was examined using a microplate reader (Thermo Multiskan MK 3) at the wavelength of 450 nm. The same method was used to detect the effect of only US on cell viability (0, 0.5, 1.0, and 1.5 W/cm^2, 1 MHz, 50% duty cycle).

After, the cytotoxicity of nanoparticles was tested on 4T1 cells by the same CCK-8 assay. In brief, the cells were seeded in 96-well plates with the density of 8000 cells per well and grown in 5% CO_2 at 37 °C overnight. The cells were then co-incubated with different concentrations of nanoparticles. After 4 h, the cells were treated with US irradiation (1 MHz, 50% duty cycle, 1.0 W/cm^2), and then continued incubated for 20 h. Finally, the CCK-8 assay was carried out to examine the cytotoxicity effect.

Measurement of intracellular CO release: The CO probe, FL-CO-1, tested the intracellular generation of CO. 4T1 cells were seeded in 6-well plates for 12 h. Then, the cells were treated using the following: (1) Control, (2) US, (3) BiOBr, (4) BiOBr + US, (5) Bi_2S_3, (6) Bi_2S_3 + US, (7) BiOBr@Bi_2S_3, (8) BiOBr@Bi_2S_3 + US. The final concentration of nanoparticles was 250 μg/mL. After that, the CO probe and $PdCl_2$ with the final 1 μM concentration were added to the above-treated cells. After 30 min incubation, the treated 4T1 cells were washed three times with PBS. Then, the cells were irradiated by US for 5 min (1 MHz, 50% duty cycle, 1.0 W/cm^2). Last, the intracellular CO production was determined using fluorescence microscope.

Measurement of intracellular ROS generation: The dihydroethidium (DHE) probe was used to test the intracellular generation of $\bullet O_2^-$ through fluorescence microscope. The •OH probe was used to test the intracellular generation of •OH and the DCFH-DA was used to test the intracellular generation of ROS. 4T1 cells were seeded into 6-well plates for 12 h (37°C, 5% CO_2). Then, the cells were treated with BiOBr, Bi_2S_3, and BiOBr@Bi_2S_3. The final concentration of nanoparticles was 250 μg/mL. Afterward, DHE, •OH probe, or DCFH-DA was added to the above-treated cells. After 1 h incubation, the treated 4T1 cells were washed thrice with PBS. Then, the cells were irradiated by US for 5 min (1 MHz, 50% duty cycle, 1.0 W/cm^2). Last, the intracellular ROS production was determined using fluorescence microscope.

Detection of intracellular mitochondrial damage: Mito-Tracker Red CMXRos

(mitochondrial red fluorescent probe), also known as MitoTracker Red CMXRos, is a derivative of Chloromethyl X-rosamine (CMXRos) with cellular permeability. It can specifically label bioactive mitochondria in cells and detect mitochondrial membrane potential. Because the Mito-Tracker Red CMXRos aggregation in mitochondria is dependent on mitochondrial membrane potential, it can also be used as an indicative probe of mitochondrial membrane potential to detect apoptosis by detecting changes in mitochondrial membrane potential. 4T1 cells were seeded into 6-well plates for 12 h (37°C, 5% CO_2). After the cells were co-incubated with the nanoparticles for 4 h, the cells were treated with US irradiation. After incubating for 12 h, 1 mL working solution of Mito-Tracker Red CMXRos was added to each well and incubated at 37°C for 30 min. Afterward, the cells were washed with PBS for three times before fluorescence images by a fluorescence microscope.

Detection of intracellular level of ATP: The intracellular level of ATP was detected by ATP assay kits. Similar to mitochondrial integrity assay detection, 4T1 cells were irradiated with US (1.0 MHz, 50% duty cycle, 1.0 W/cm^2, 5 min). After 12 h, 4T1 cells were lysed and the supernatant was immediately collected by centrifugation for detection of ATP level in vitro by assay kits (Beyotime).

Intracellular DNA damage measurement: The phosphorylated H2AX (γ-H2AX) is the marker of early DNA damage. The H2AX Phosphorylation Assay Kit was used to detect intracellular DNA damage. 4T1 cells were planted into 12-well plates at the density of 3×10^6 cells per dish for 12 h at 37 °C in the dark and then co-incubated with BiOBr, Bi_2S_3, or BiOBr@Bi_2S_3 (250 μg/mL) for another 4 h. Then, the cells were treated with or without US irradiation (1.0 MHz, 50% duty cycle, 1.0 W/cm^2, 5 min). After incubation for another 6 h, 4T1 cells were stained with the H2AX Phosphorylation Assay Kit. After incubation for another 1 h, excessive dyes were removed by washing three times with PBS, and then 4T1 cells were observed on a fluorescence microscope.

Glucose and lactate assay: 4T1 cells were seeded into wells of a 6-well plate and divided into 8 groups [1) Control, 2) US, 3) BiOBr, 4) BiOBr + US, 5) Bi_2S_3, 6) Bi_2S_3 + US, 7) BiOBr@Bi_2S_3, 8) BiOBr@Bi_2S_3 + US.]. After 12 h culture, cells were treated with BiOBr, Bi_2S_3, and BiOBr@Bi_2S_3 (250 μg/mL) and irradiated with US (1.0 MHz, 50% duty cycle, 1.0 W/cm^2, 5 min). Then, glucose concentration and lactate concentration in the treated cells were detected by Glucose Assay Kit with O-toluidine (Beyotime Biotech. Inc.) and Lactic Acid assay kit (A019-2-1, NanJing JianCheng Bioengineering Institute, Nanjing, China), respectively.

Cell proliferation assay: The BeyoClick™ EdU Cell Proliferation Kit with Alexa Fluor 555

was used to evaluate the proliferative capacity of 4T1 cells after different treatments. EdU(5-ethynyl-2'-deoxyuridine) is a novel thymidine analog. EdU can be incorporated into newly synthesized DNA in place of thymidine during DNA synthesis. On the other hand, the ethynyl group on EdU can covalently react with the fluorescently labeled small molecule azide probe (Azide Alexa Fluor 555) through the catalysis of Cu^+ to form a stable triazole ring. It's called a click response. Through a click reaction, newly synthesized DNA is labeled with an Azide Alexa Fluor 555 fluorescent probe, allowing proliferating cells to be detected using appropriate fluorescent detection equipment. 4T1 cells were planted into 6-well plates at the density of 10^5 cells per well for 12 h at 37 °C in the dark and then co-incubated with BiOBr, Bi_2S_3, or BiOBr@Bi_2S_3 (250 µg/mL) for another 4 h. Then, the cells were treated with or without US irradiation (1.0 MHz, 50% duty cycle, 1.0 W/cm^2, 5 min). After incubation for another 12 h, the cells were stained according to product instructions. The proliferation of cells in each group was then detected.

Annexin V/PI assay: 4T1 cells were seeded in 6-well plates at the density of 2×10^5 cells per well and allowed to adhere for 12 h. Then the cells were incubated with BiOBr, Bi_2S_3, or BiOBr@Bi_2S_3 (250 µg/mL) for 4 h. After that, the cells were treated with US irradiation (1 MHz, 50% duty cycle, 1.0 W/cm^2, 5 min) incubation for another 12 h before stained with 5 µL Annexin V-FITC for 15 min and 5 µL PI for 5 min in the dark. The fluorescence intensity of cells was detected by flow cytometry. The apoptosis of cells in each group was then detected.

Tumor Model: The mice used in the animal experiments were female Balb/c mice aged 6-8 weeks. They were purchased from the Changchun Institute of Biological Products and all the mice were handled under the protocol approved by the Institutional Animal Care and Use Committee of Jilin University (approval number: SCXK (Liao) 2020-0001).

In vivo biocompatibility of BiOBr@Bi_2S_3 nanocomposites: Healthy female Balb/c mice were intravenously injected with BiOBr@Bi_2S_3 nanocomposites (10 mg/kg, 100 µL). At the indicated time, the mice were euthanized and their blood samples were collected to perform blood panel analysis and blood biochemistry assay. The major organs containing heart, liver, spleen, lung, and kidney were harvested for hematoxylin and eosin (H&E) staining.

In vivo biodistirbution of BiOBr@Bi_2S_3 nanocomposites: In the quantitative biodistribution analysis, 4T1 tumor-bearing mice were intravenously injected with BiOBr@Bi_2S_3 nanoparticles (10 mg/kg 100 µL,). At 8, 12, 24, and 48 h post-administration, the mice were sacrificed. The tumor tissues and main organs containing heart, liver, spleen, lung, and kidney were excised and weighted, then digested in aqua regia under heat treatment

(70 °C) for homogenate to analyze the content of Bi ions in the samples using Inductively coupled plasma-Mass Spectrometry (ICP-MS). To further investigate how nanoparticles were metabolized in the body, mouse feces were collected at fixed points in time to detect Bi levels (0 h, 12 h, 1 D, 2 D, 3 D, 5 D, and 7 D).

Tumor suppression experiments in vivo: To develop a tumor model, 4T1 breast cancer cells suspended in PBS was subcutaneously injected into the right leg of each female Balb/c mice. When the tumor volume reached around 100 mm^3, mice were randomly divided into eight groups consisting of five mice in each group: (1) Control, (2) US, (3) BiOBr, (4) BiOBr + US, (5) Bi_2S_3 (6) Bi_2S_3 + US, (7) BiOBr@Bi_2S_3 and (8) BiOBr@Bi_2S_3 + US. Then, the mice were received 100 μL of intravenous administration of BiOBr, Bi_2S_3 or BiOBr@Bi_2S_3 nanocomposites (10 mg/kg), respectively. In US irradiation groups, the tumors were treated by US irradiation (1.0 MHz, 50% duty cycle, 1.0 W/cm^2, 10 min) after the intravenous injection of 12 h, 24 h, and 48 h. During the treatments, tumor size and body weight were monitored every two days. The tumor volume was calculated by the following formula: volume = length × $width^2/2$. After 14 days of treatment, all the mice were humanely executed. Main organs (heart, liver, spleen, lung, and kidney) and tumors were extracted for histological analysis by the H&E staining method staining analysis.

CO, DHE, •OH, DCFH-DA, γ-H2AX, Caspase-3, and Ki-67: The collected tumors were fixed with 10% paraformaldehyde, embedded in paraffin, sectioned into ~4 μm, and stained with FL-CO-1 probe to detect the production of CO in the tumor, DHE to evaluate the $•O_2^-$ generation, •OH probe to evaluate the •OH generation, DCFH-DA to evaluate the ROS generation, γ-H2AX to determine DNA damage, Caspase-3 to determine cellular apoptosis, and Ki-67 antibody staining to determine the growth fraction of cells.

Statistical analysis: Regular statistical tests were performed using Excel 2016 and Origin 2020. Significance analysis were conducted using one-way analysis of variance (ANOVA) in GraphPad Prism 7. The images and fluorescence intensity were processed or analyzed by Adobe Photoshop 2015 and Image J software. The animals and cells experiments were performed at least three times, and the results are expressed as mean ± S.D. In all cases, a p value less than 0.05 was considered to statistically significant, *$p< 0.05$, **$p< 0.01$, ***$p< 0.001$, ****$p< 0.0001$., and ns: no significant difference.

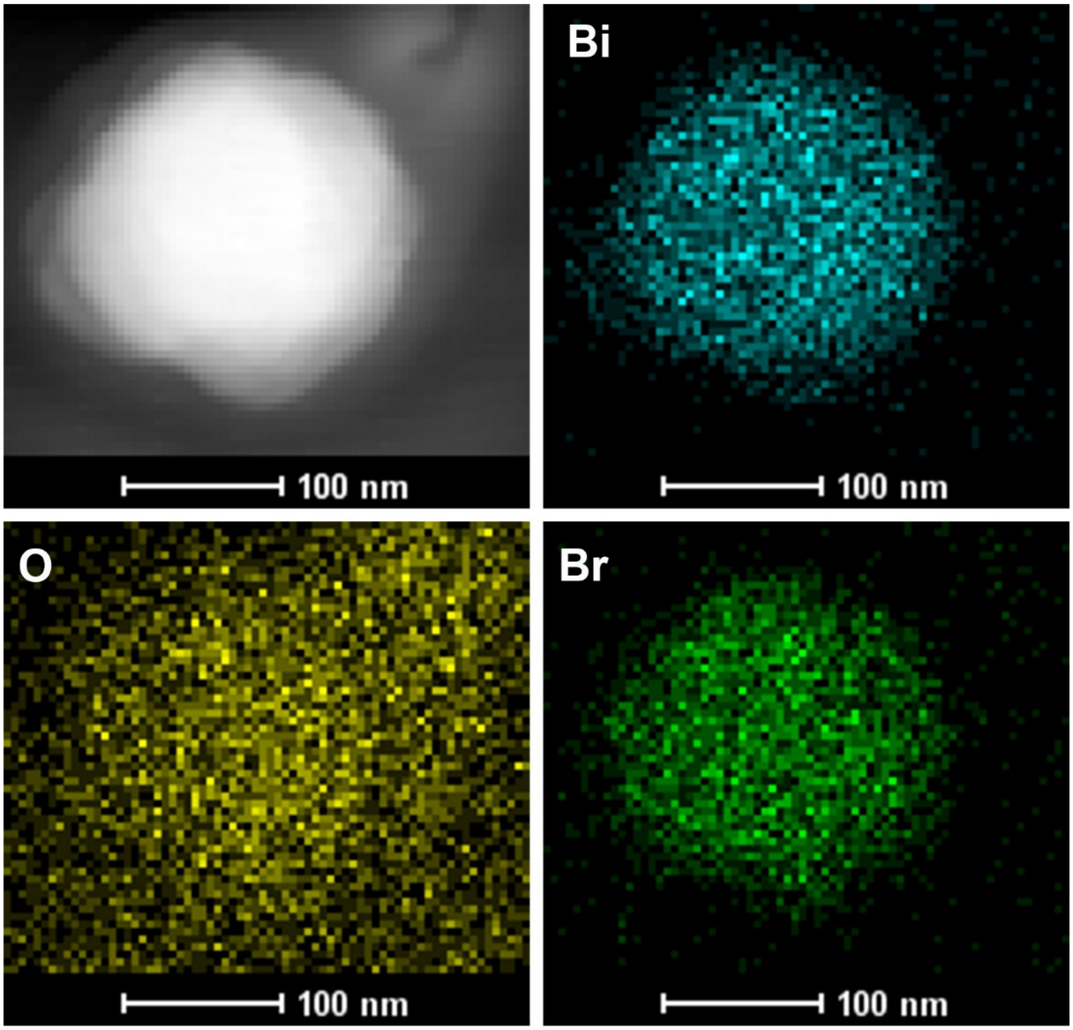

Figure S1. HAADF image of BiOBr nanosheets and the corresponding elemental mapping images.

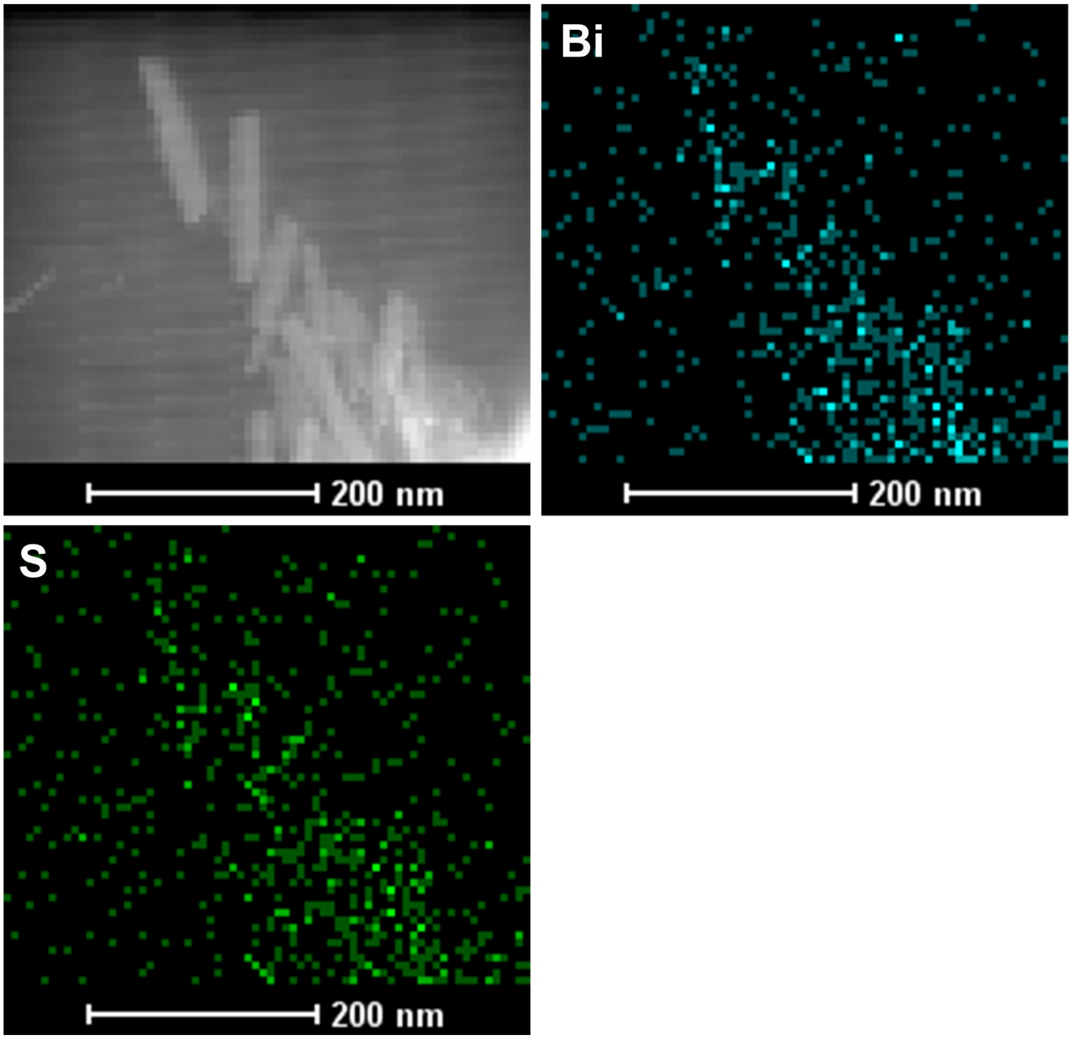

Figure S2. HAADF image of Bi_2S_3 nanorods and the corresponding elemental mapping images.

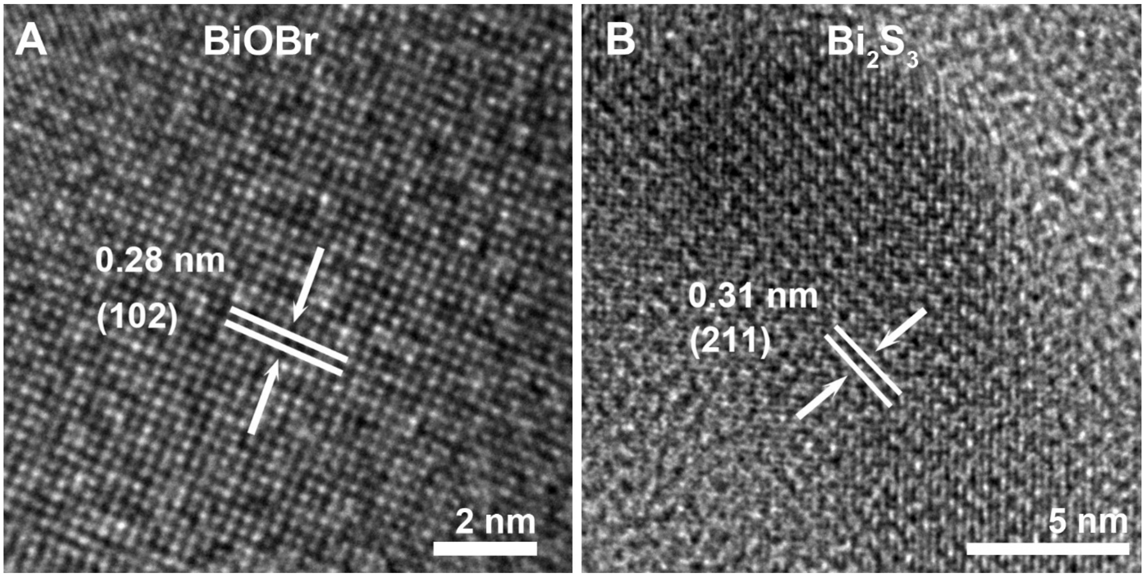

Figure S3. A, B) HRTEM images of the BiOBr (A) and Bi_2S_3 (B).

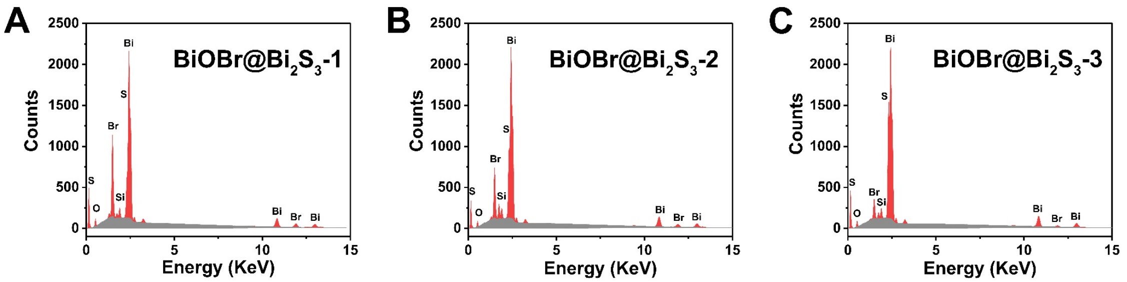

Element Series	unn. C (wt.%)	norm. C (wt.%)	Atom. C (at.%)	Error (%)
Oxygen K-series	11.12	12.81	54.02	2.4
Silicon K-series	0.37	0.43	1.03	0.1
Sulfur K-series	3.78	4.35	9.16	0.2
Bromine K-series	15.31	17.64	14.89	0.7
Bismuth L-series	56.23	64.77	20.91	2.2

Element Series	unn. C (wt.%)	norm. C (wt.%)	Atom. C (at.%)	Error (%)
Oxygen K-series	11.60	13.24	52.29	2.7
Silicon K-series	1.32	1.51	3.39	0.1
Sulfur K-series	6.58	7.51	14.80	0.3
Bromine K-series	10.79	12.32	9.74	0.5
Bismuth L-series	57.30	65.42	19.77	2.2

Element Series	unn. C (wt.%)	norm. C (wt.%)	Atom. C (at.%)	Error (%)
Oxygen K-series	9.74	11.49	46.15	2.3
Silicon K-series	0.5	0.59	1.35	0.1
Sulfur K-series	11.41	13.45	26.96	0.5
Bromine K-series	4.50	5.30	4.27	0.3
Bismuth L-series	58.69	69.17	21.28	2.2

Figure S4. The EDS analysis of BiOBr@Bi_2S_3-1 (A), BiOBr@Bi_2S_3-2 (B), and BiOBr@Bi_2S_3-3 (C).

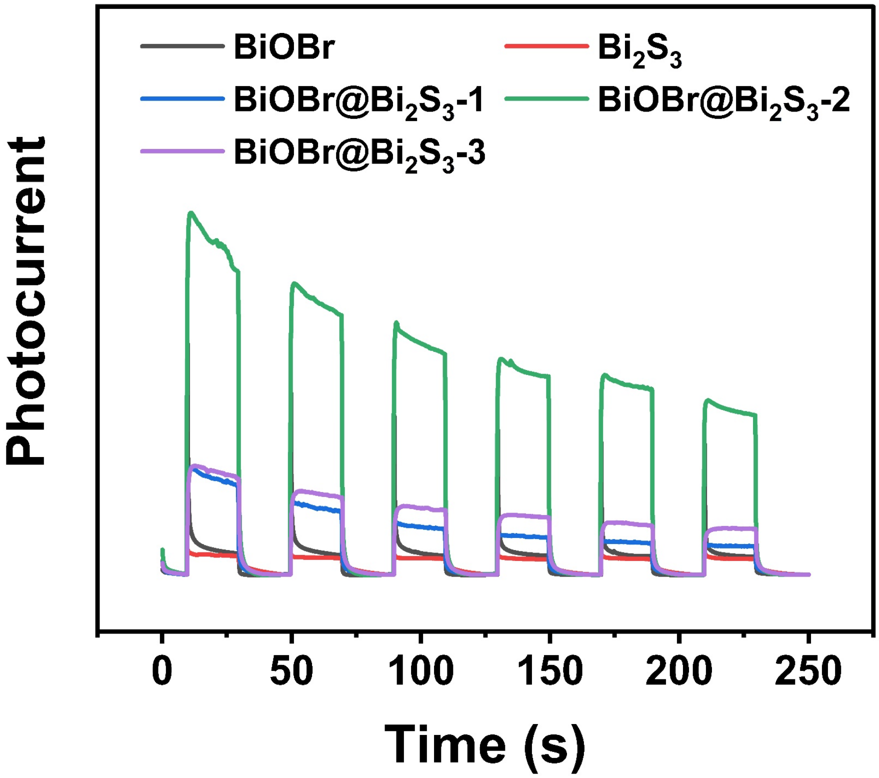

Figure S5. Transient photocurrent spectra of BiOBr, Bi_2S_3, BiOBr@Bi_2S_3-1, BiOBr@Bi_2S_3-2, and BiOBr@Bi_2S_3-3.

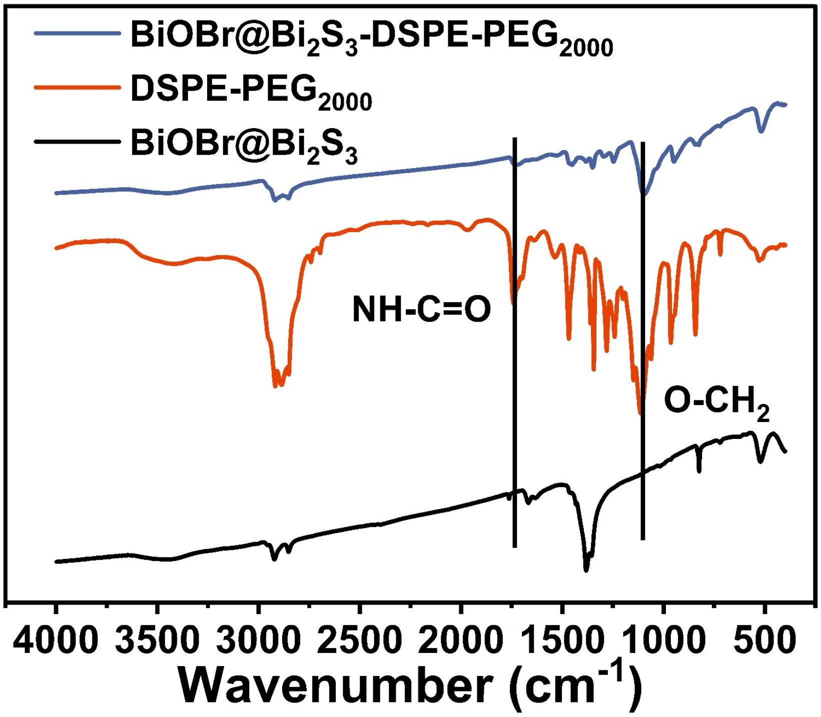

Figure S6. FT-IR spectra of the BiOBr, DSPE-PEG_{2000}, and BiOBr@Bi_2S_3-DSPE-PEG_{2000}.

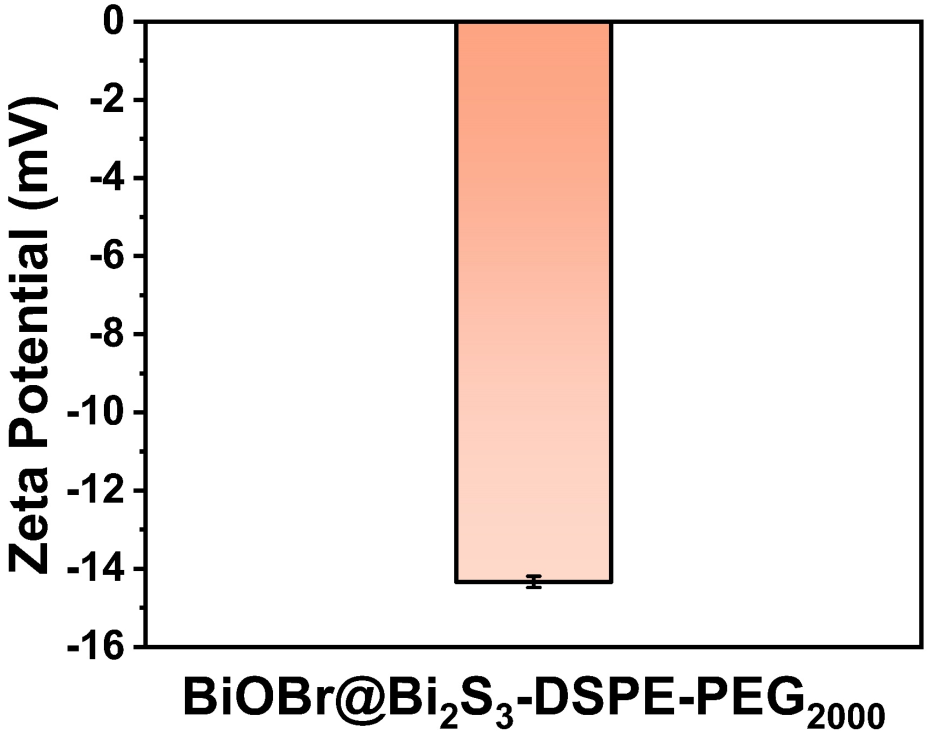

Figure S7. Zeta potential (Mean ± SD, n = 3) of BiOBr@Bi_2S_3-DSPE-PEG_{2000}.

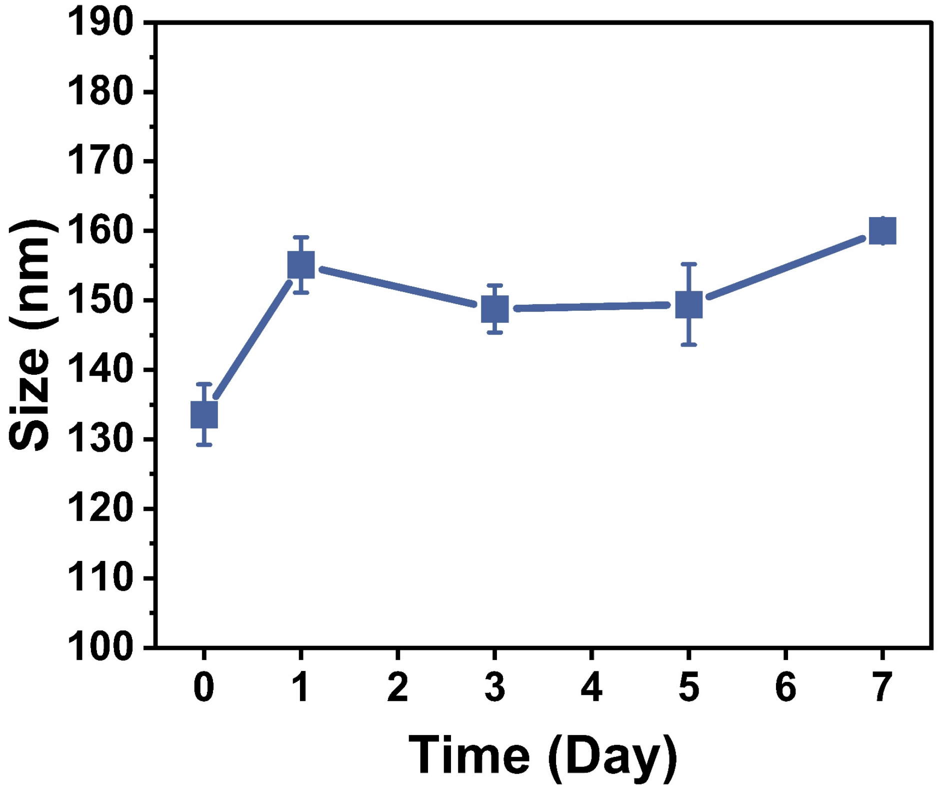

Figure S8. Hydrodynamic diameters (Mean ± SD, n = 3) of BiOBr@Bi_2S_3-DSPE-PEG_{2000} in fetal bovine serum for 7 days.

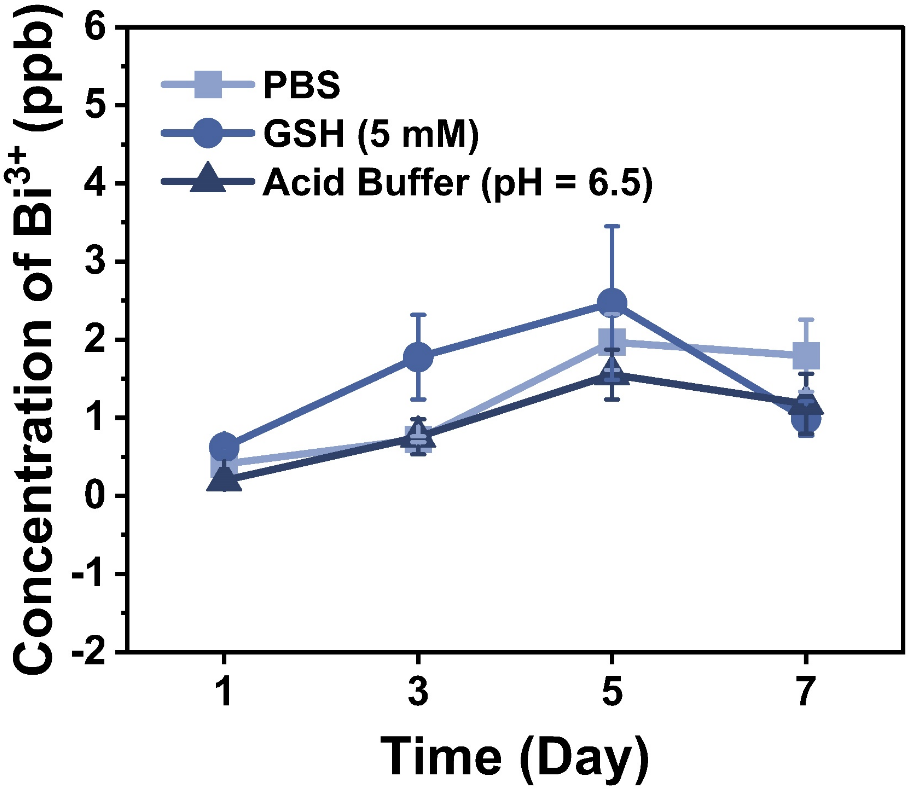

Figure S9. Evaluation of degradability of nanoparticles (Mean ± SD, n = 3).

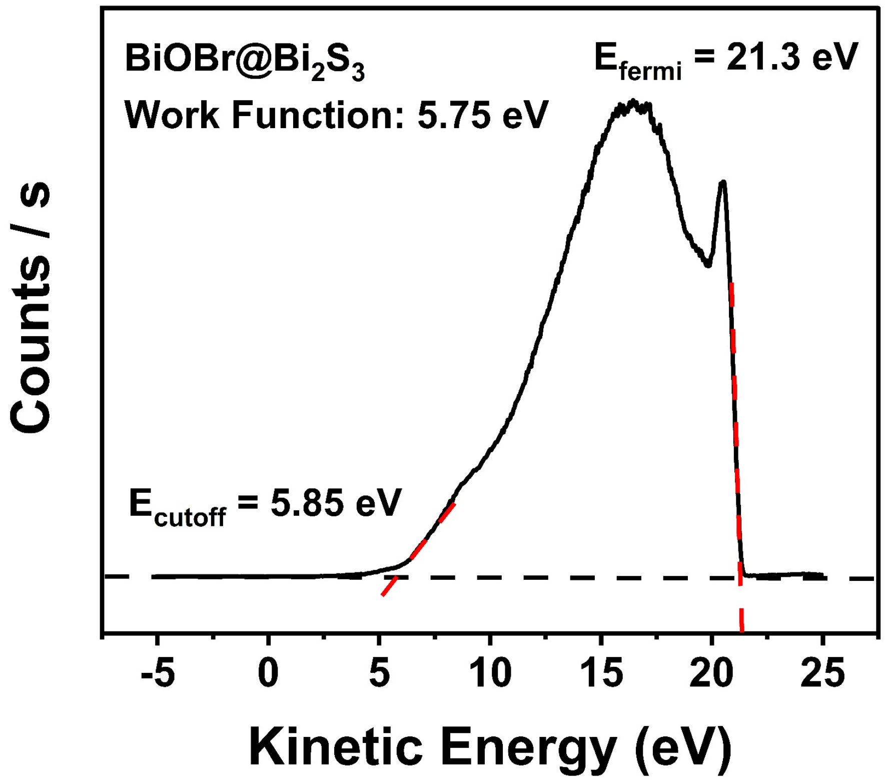

Figure S10. The W_f of $BiOBr@Bi_2S_3$.

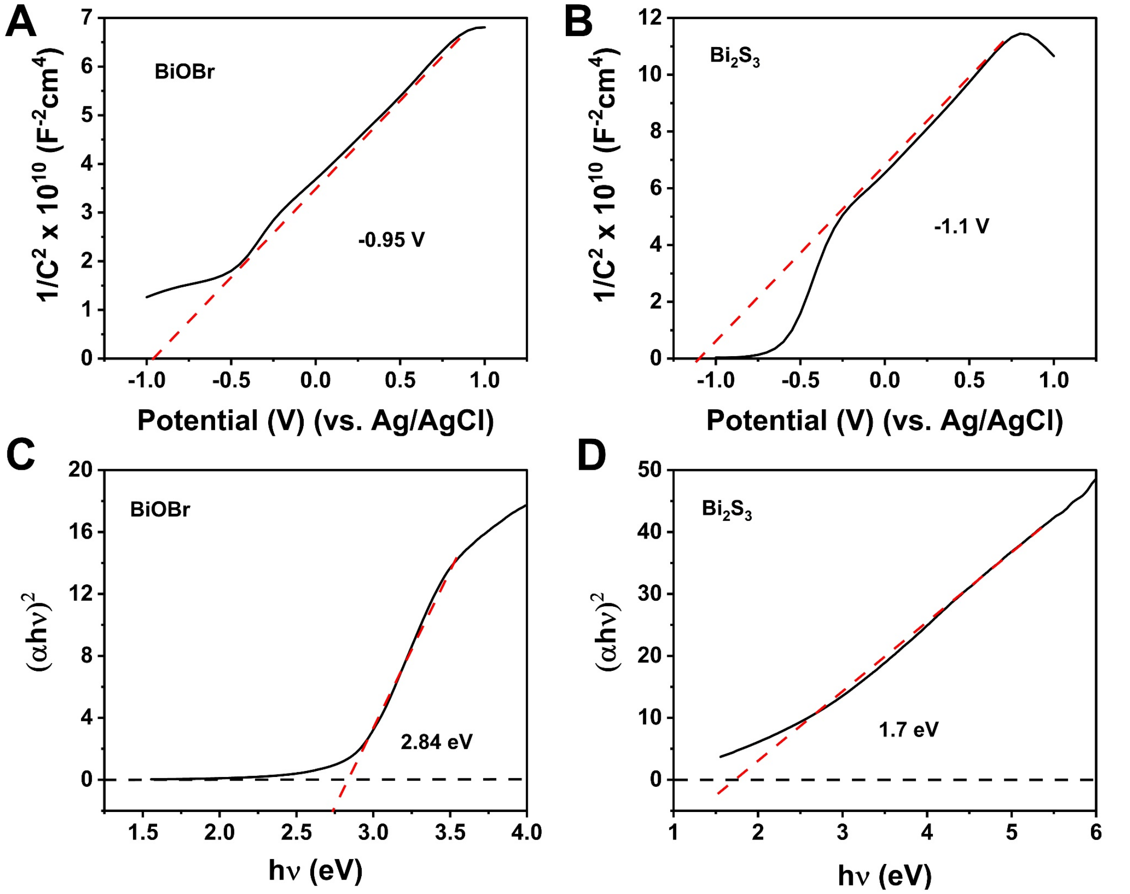

Figure S11. Mott-Schottky plots of A) BiOBr and B) Bi_2S_3. The bandgaps of C) BiOBr and D) Bi_2S_3.

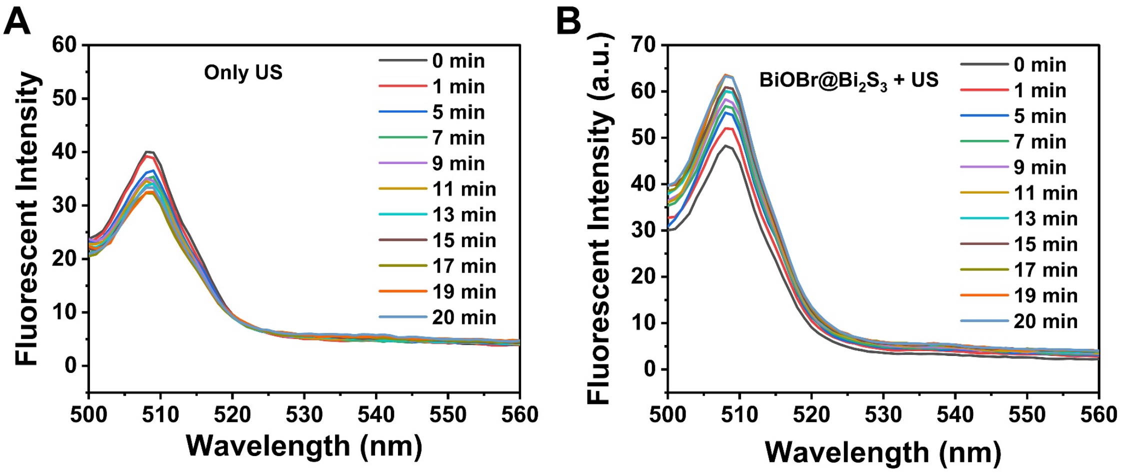

Figure S12. Fluorescence spectra changes of the probe (5 μM FL-CO-1 + 5 μM $PdCl_2$) in H_2O (A) and BiOBr@Bi_2S_3 solutions (250 μg/mL, B) under US irradiation.

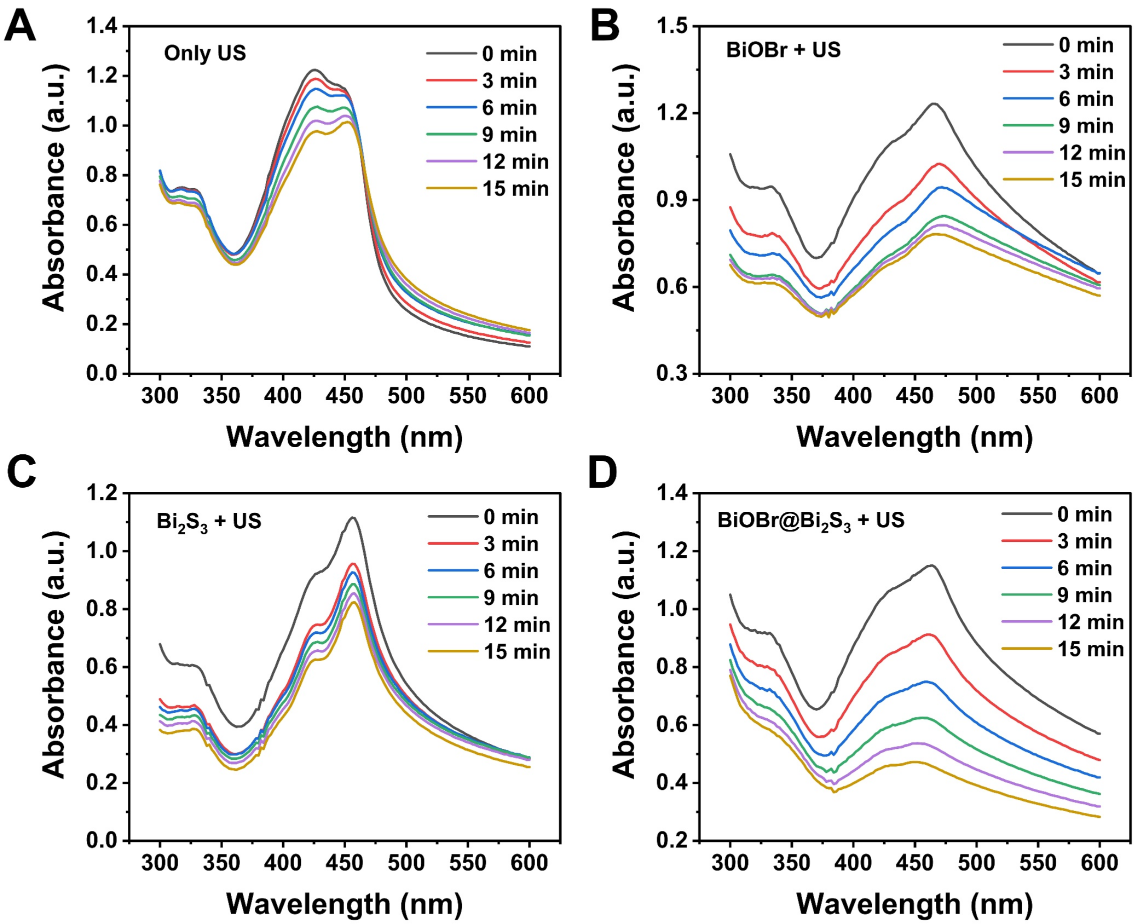

Figure S13. Time-dependent DPBF consumption by A) H_2O, B) BiOBr solution, C) Bi_2S_3 solution, and D) BiOBr@Bi_2S_3 under US irradiation.

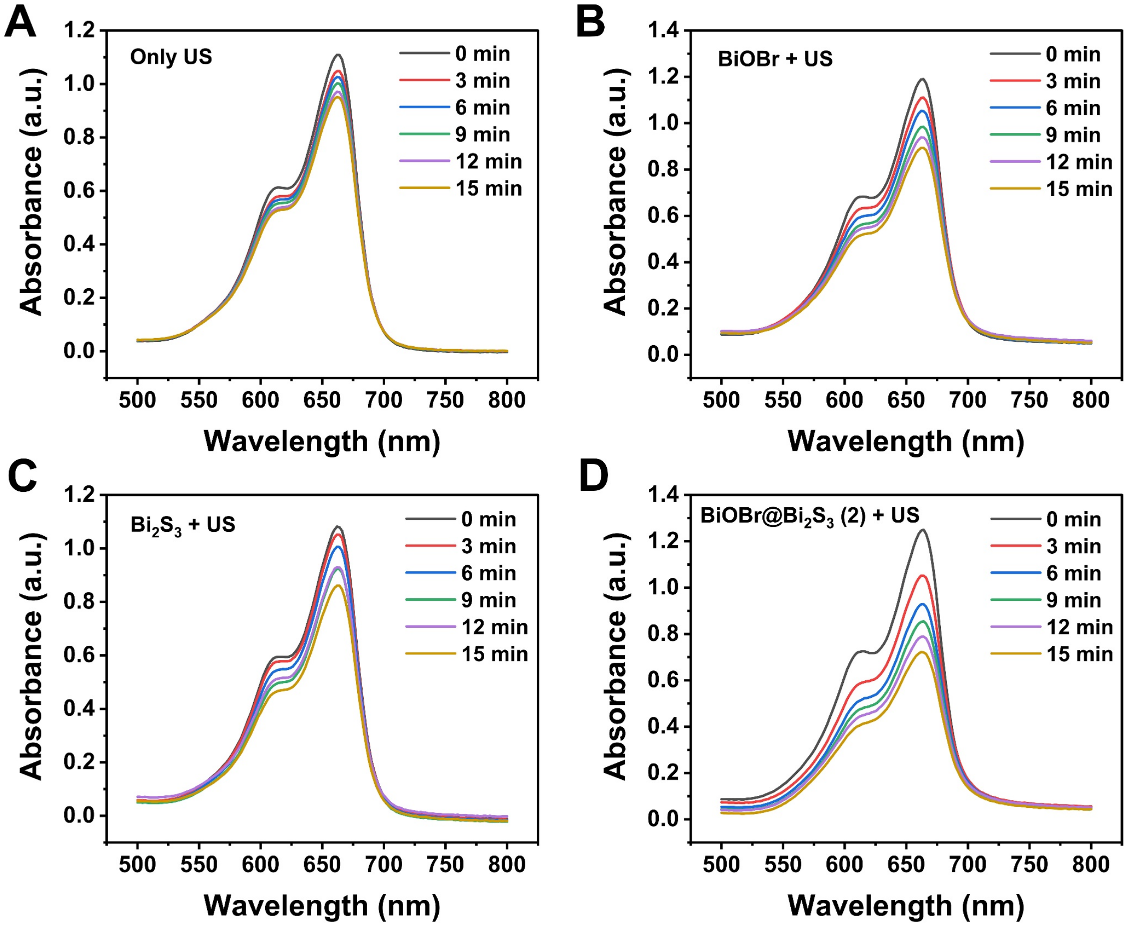

Figure S14. Time-dependent MB consumption by A) H_2O, B) BiOBr solution, C) Bi_2S_3 solution, and D) BiOBr@Bi_2S_3 under US irradiation.

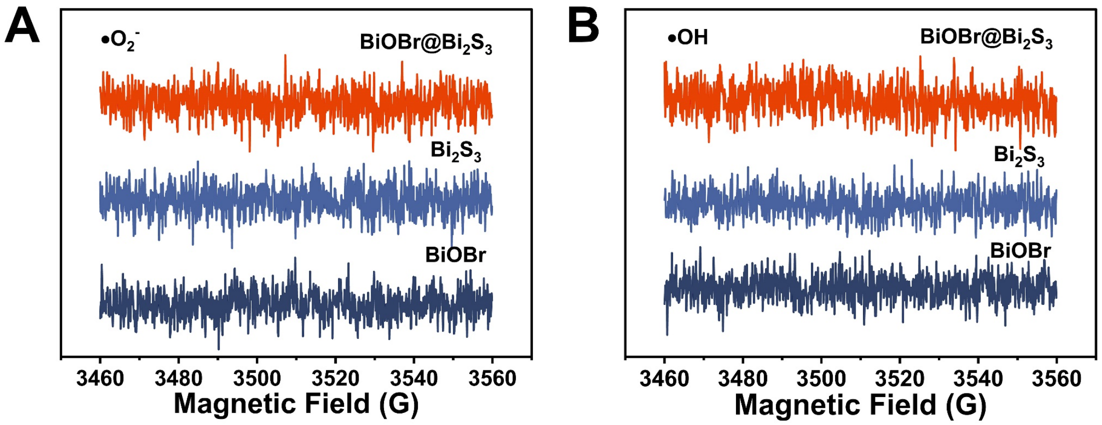

Figure S15. Comparison of •O_2^- (A) and •OH (B) generation for BiOBr, Bi_2S_3, and BiOBr@Bi_2S_3 without US irradiation demonstrated by ESR spectra.

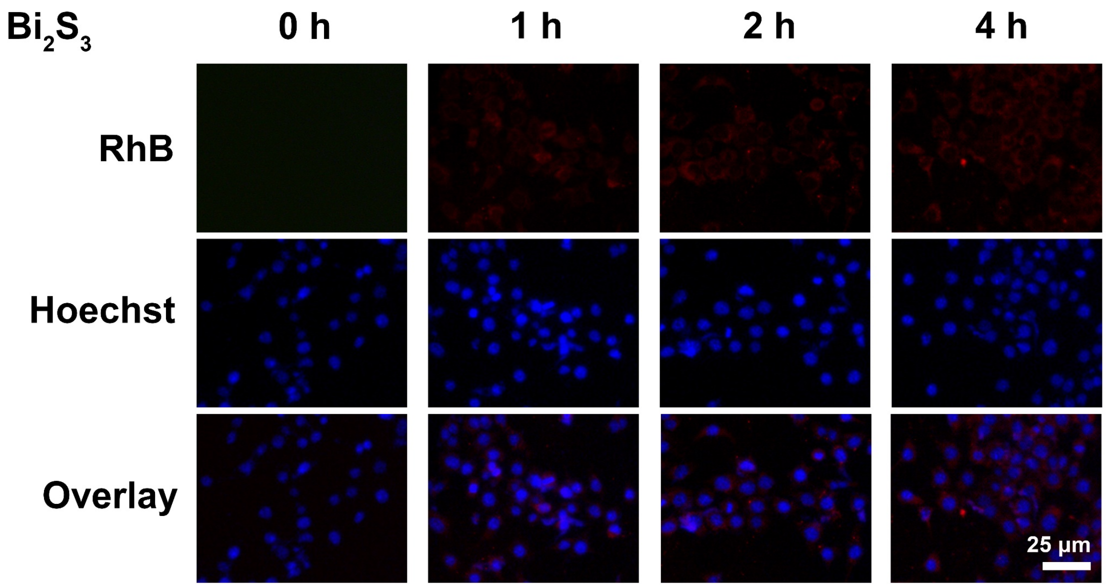

Figure S16. In vitro cellular uptake of RhB-conjugated BiOBr.

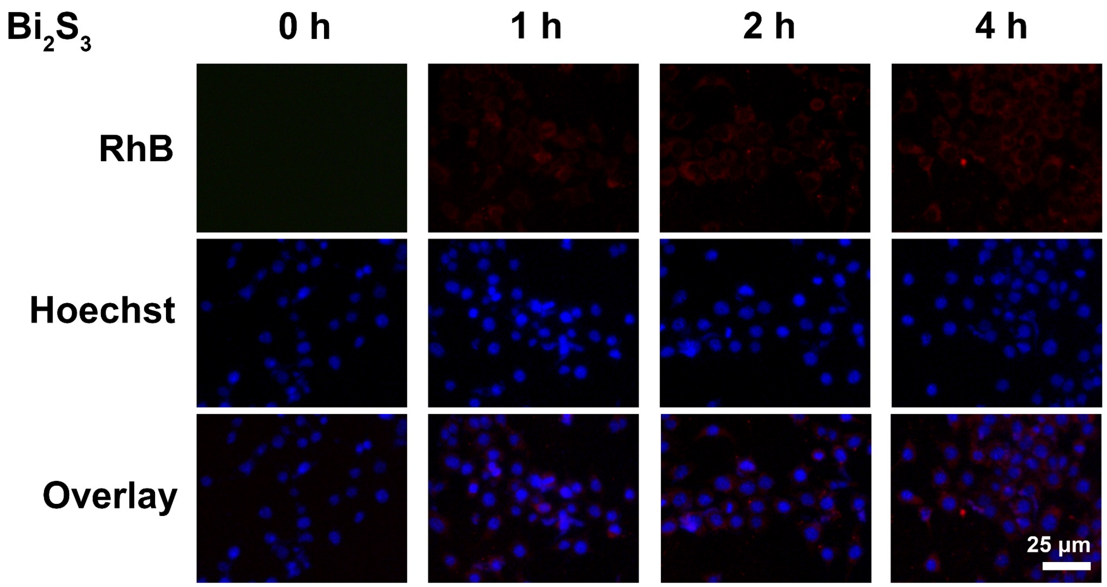

Figure S17. In vitro cellular uptake of RhB-conjugated Bi_2S_3.

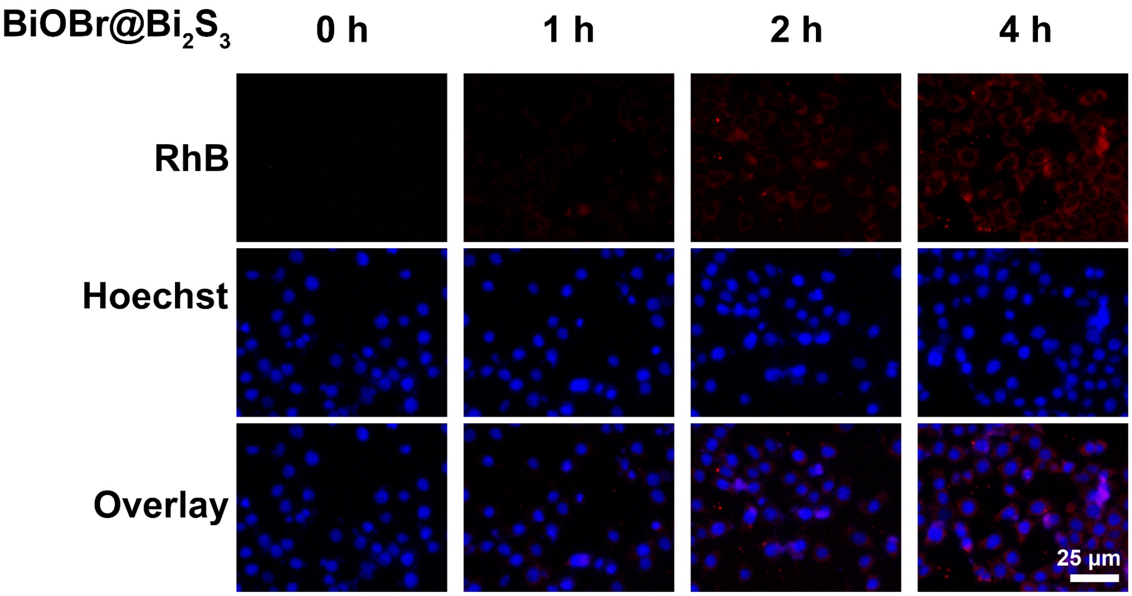

Figure S18. In vitro cellular uptake of RhB-conjugated BiOBr@Bi_2S_3.

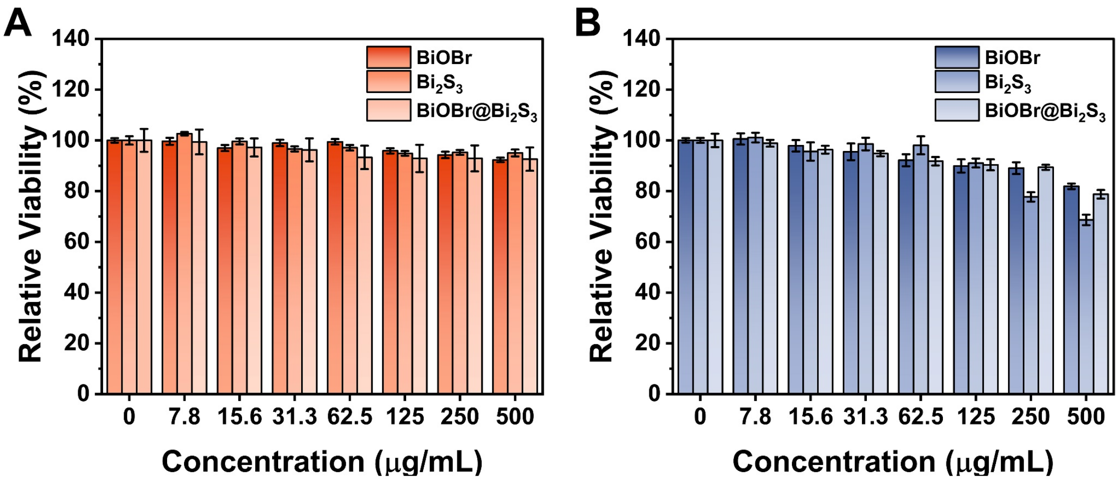

Figure S19. The biocompatibility of BiOBr, Bi_2S_3, and BiOBr@Bi_2S_3 on L929 (A) and 4T1 cells (B) (Mean ± SD, n = 5).

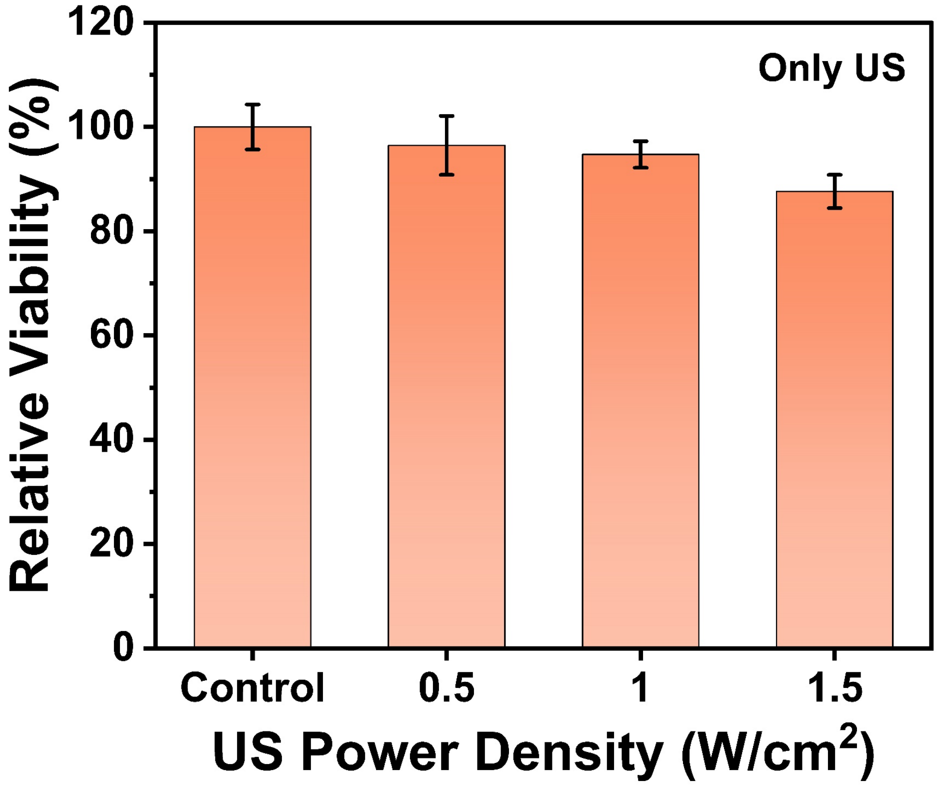

Figure S20. Cell viability of 4T1 cells at different US power density (Mean ± SD, n = 5).

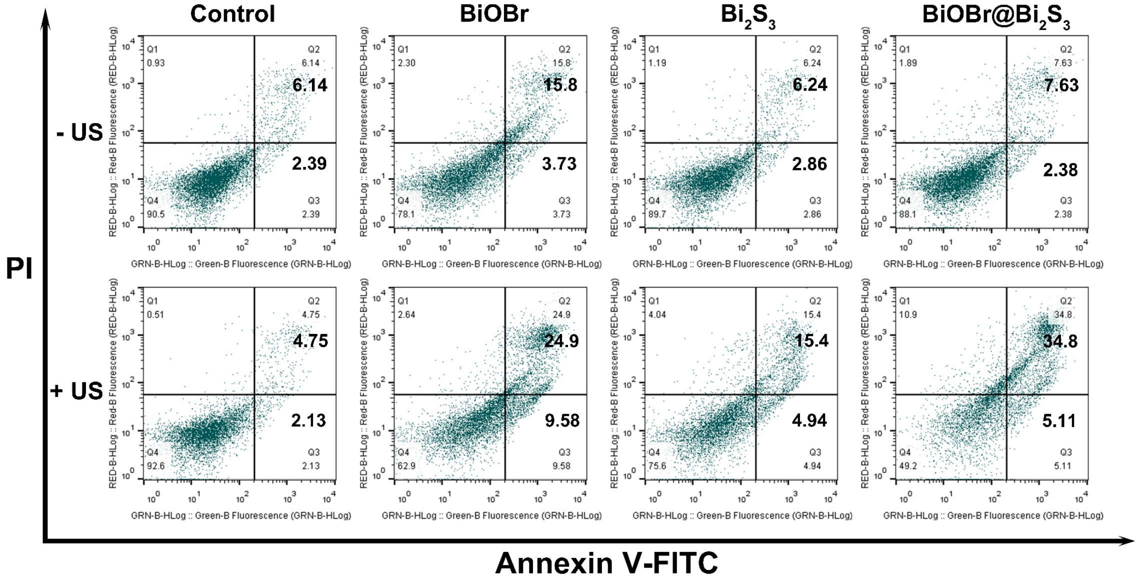

Figure S21. Apoptosis behaviors of 4T1 cells under different treatments were detected using flow cytometry.

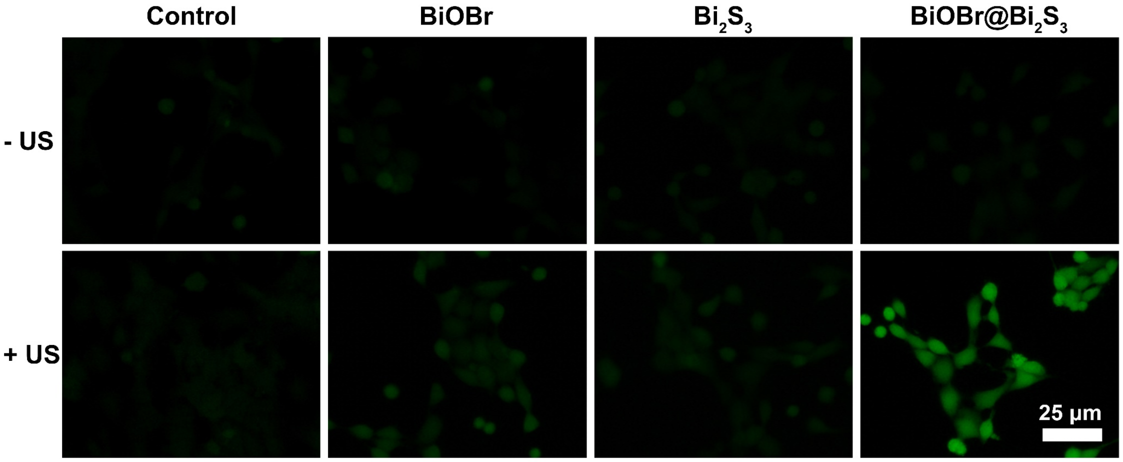

Figure S22. Detection of ROS intensity by DCFH-DA in different treatments.

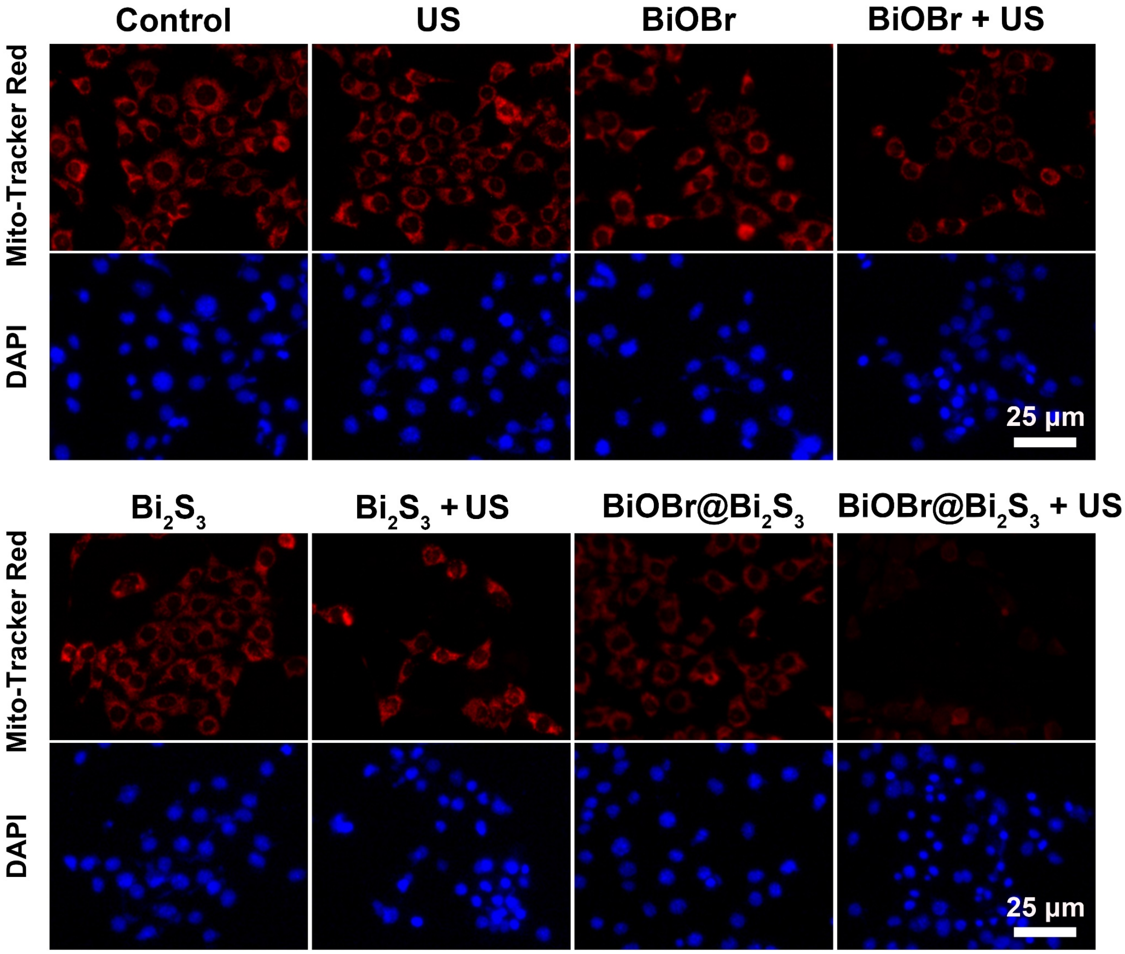

Figure S23. Mitotracker Red Probes staining images of 4T1 cells after various treatments.

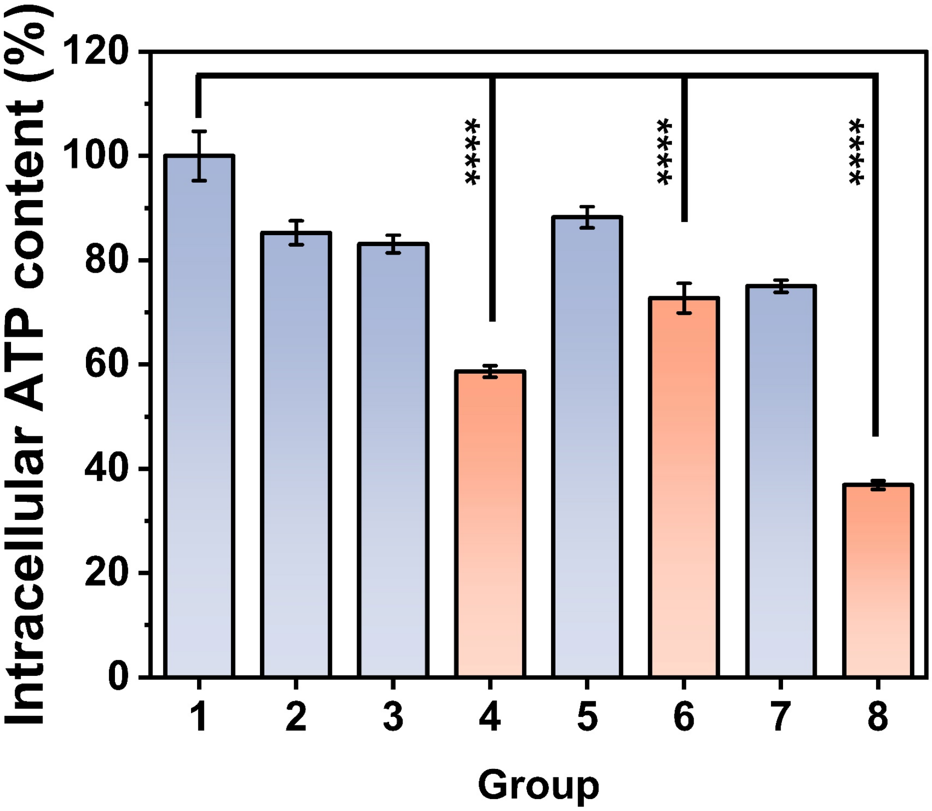

Figure S24. The intracellular ATP levels after various treatments (mean ± SD, n = 3). The *p* values were calculated by one-way analysis of variance (ANOVA), ****p < 0.0001, ***p < 0.001, **p < 0.01, and *p < 0.05. Group: 1) Control, 2) US, 3) BiOBr, 4) BiOBr + US, 5) Bi_2S_3, 6) Bi_2S_3 + US, 7) BiOBr@Bi_2S_3, 8) BiOBr@Bi_2S_3 + US.

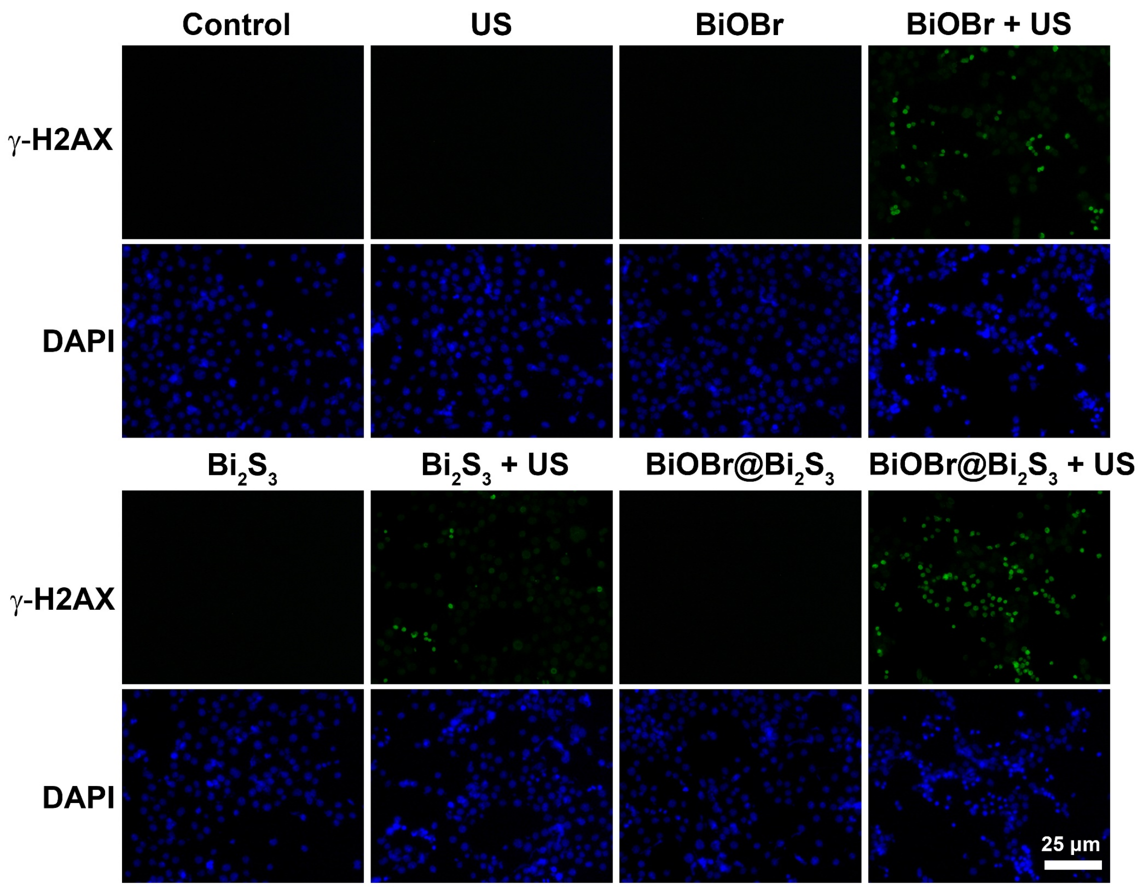

Figure S25. Early DNA damage of 4T1 cells under various treatments.

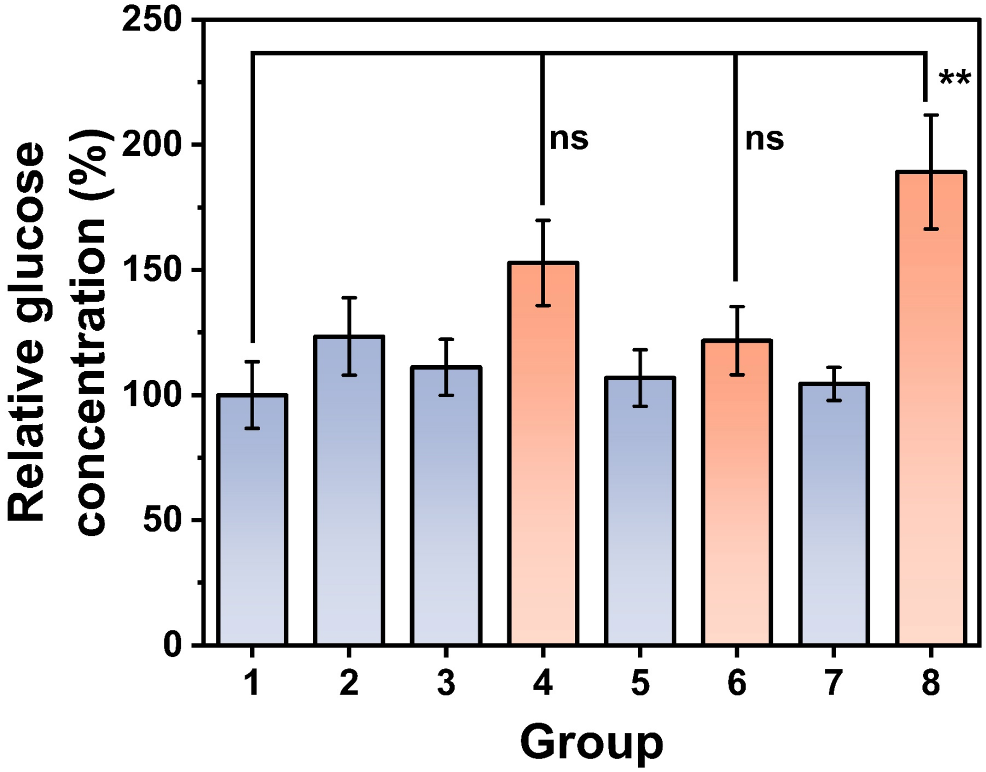

Figure S26. Relative glucose concentration in 4T1 cells with different treatments (mean ± SD, n = 3). The *p* values were calculated by one-way analysis of variance (ANOVA), ****$p < 0.0001$, ***$p < 0.001$, **$p < 0.01$, and *$p < 0.05$. Group: 1) Control, 2) US, 3) BiOBr, 4) BiOBr + US, 5) Bi_2S_3, 6) Bi_2S_3 + US, 7) BiOBr@Bi_2S_3, 8) BiOBr@Bi_2S_3 + US.

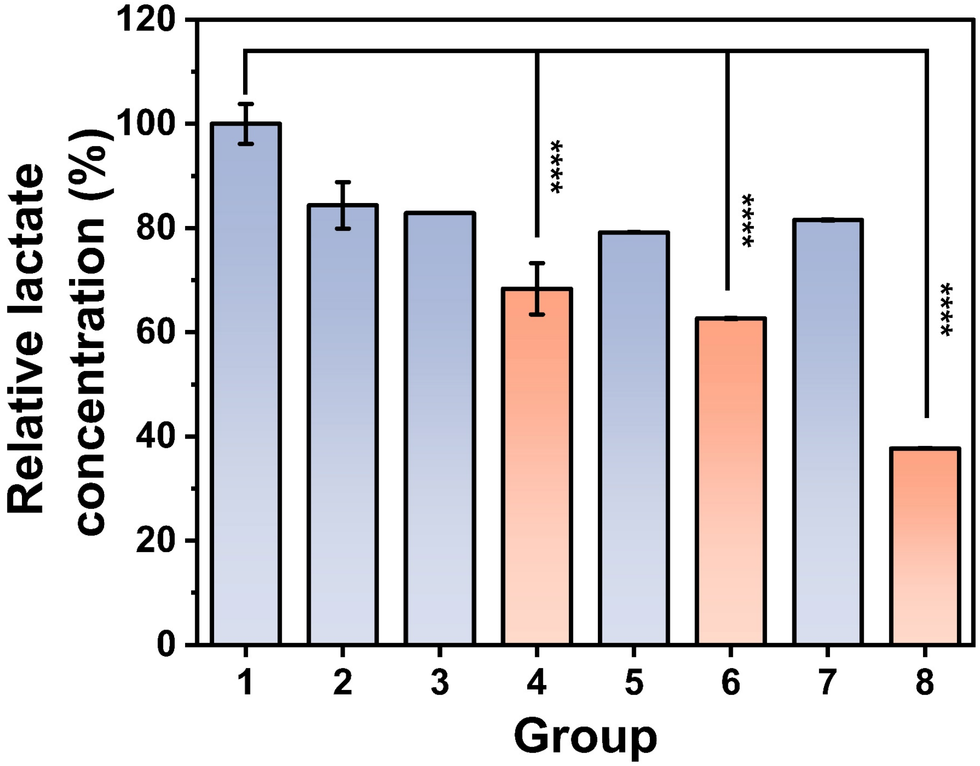

Figure S27. Relative lactate concentration in 4T1 cells with different treatments (mean ± SD, n = 3). The *p* values were calculated by one-way analysis of variance (ANOVA), ****p < 0.0001, ***p < 0.001, **p < 0.01, and *p < 0.05. Group: 1) Control, 2) US, 3) BiOBr, 4) BiOBr + US, 5) Bi_2S_3, 6) Bi_2S_3 + US, 7) BiOBr@Bi_2S_3, 8) BiOBr@Bi_2S_3 + US.

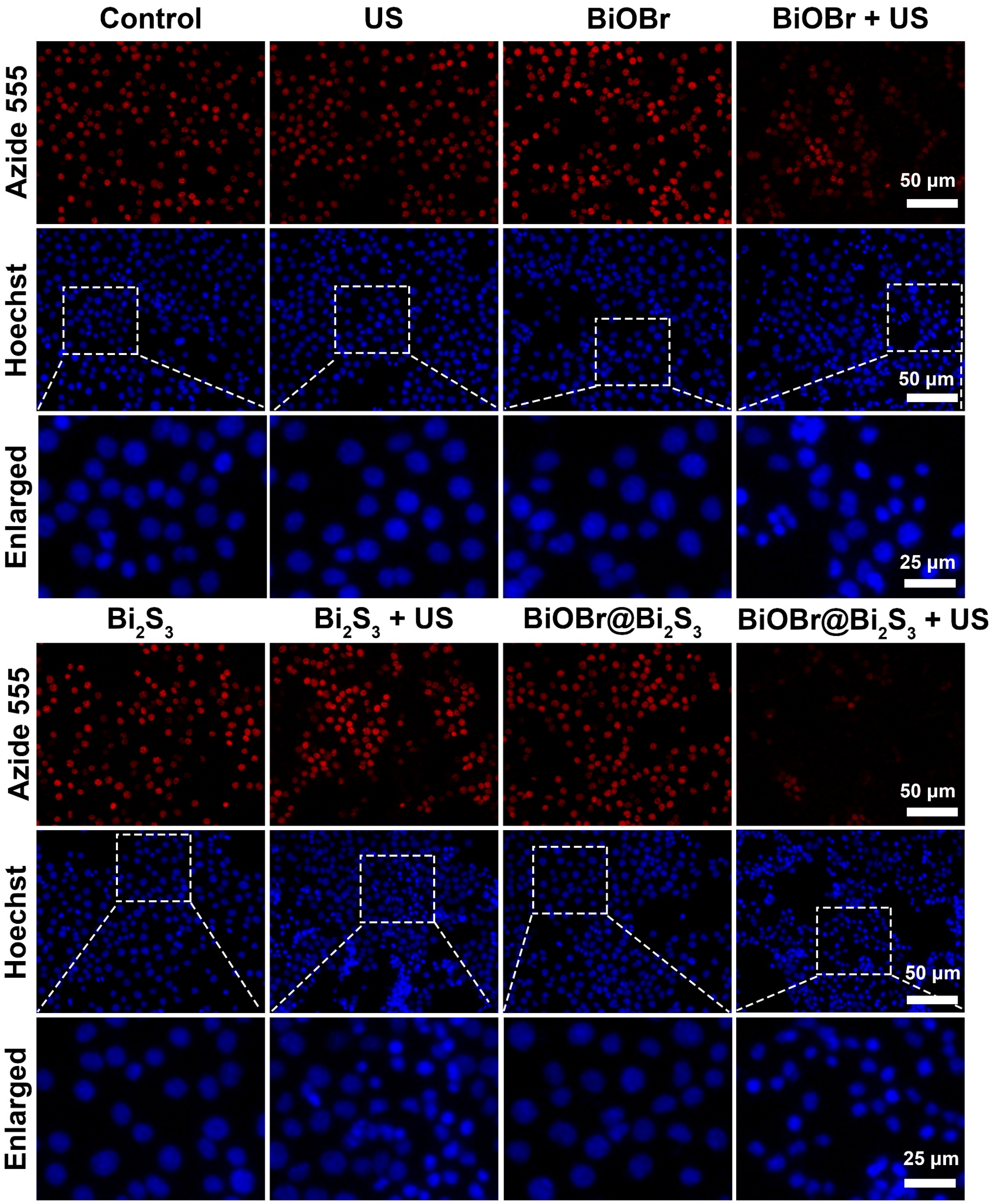

Figure S28. Fluorescence microscopy images of the reproductive capacity of 4T1 cells after various treatments.

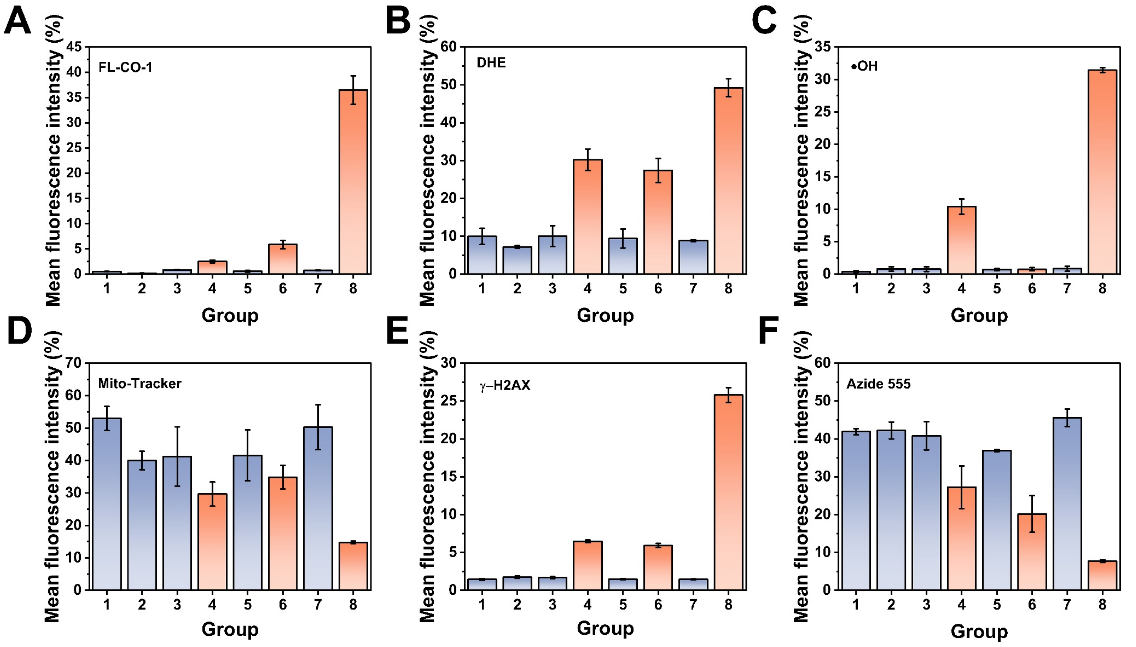

Figure S29. Analysis of mean fluorescence intensity (Mean ± SD, n = 3). Group: 1) Control, 2) US, 3) BiOBr, 4) BiOBr + US, 5) Bi_2S_3, 6) Bi_2S_3 + US, 7) BiOBr@Bi_2S_3, 8) BiOBr@Bi_2S_3 + US.

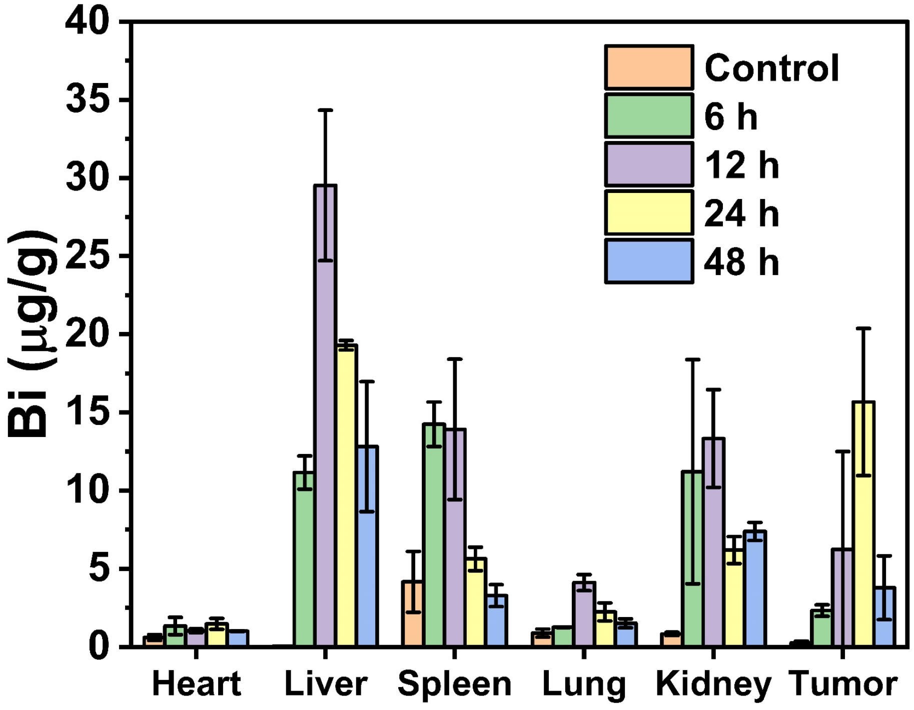

Figure S30. Bio-distribution of $BiOBr@Bi_2S_3$ in major organs and tumor (Mean ± SD, n = 3).

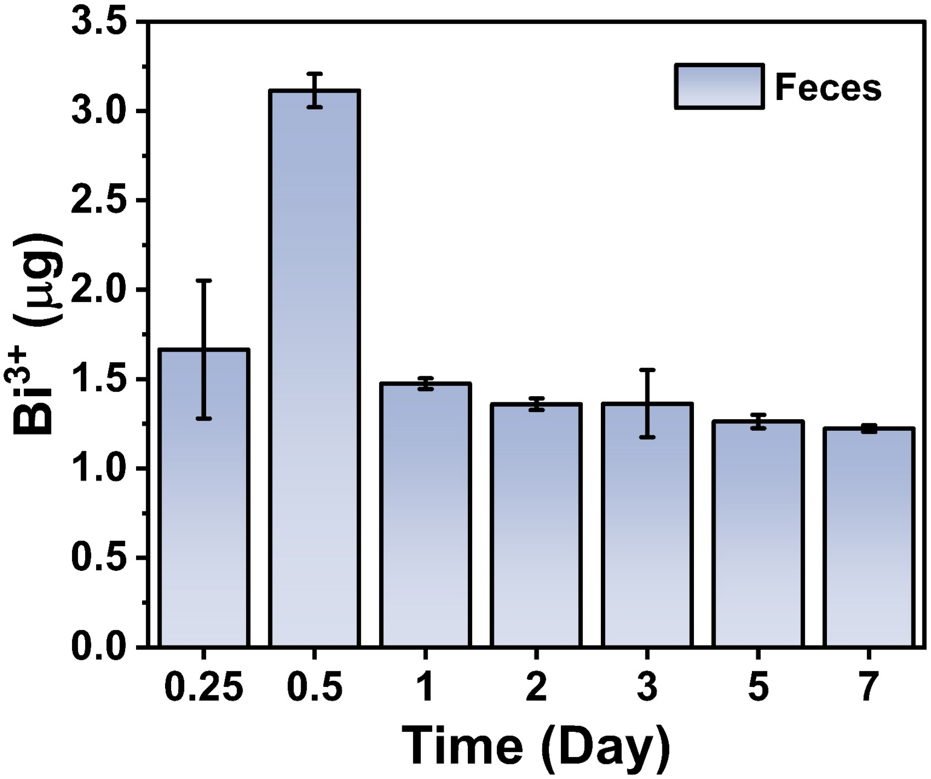

Figure S31. Bi^{3+} contents in feces of mice after intravenous injection of BiOBr@Bi_2S_3-DSPE-PEG_{2000} within 7 D (Mean ± SD, n = 3).

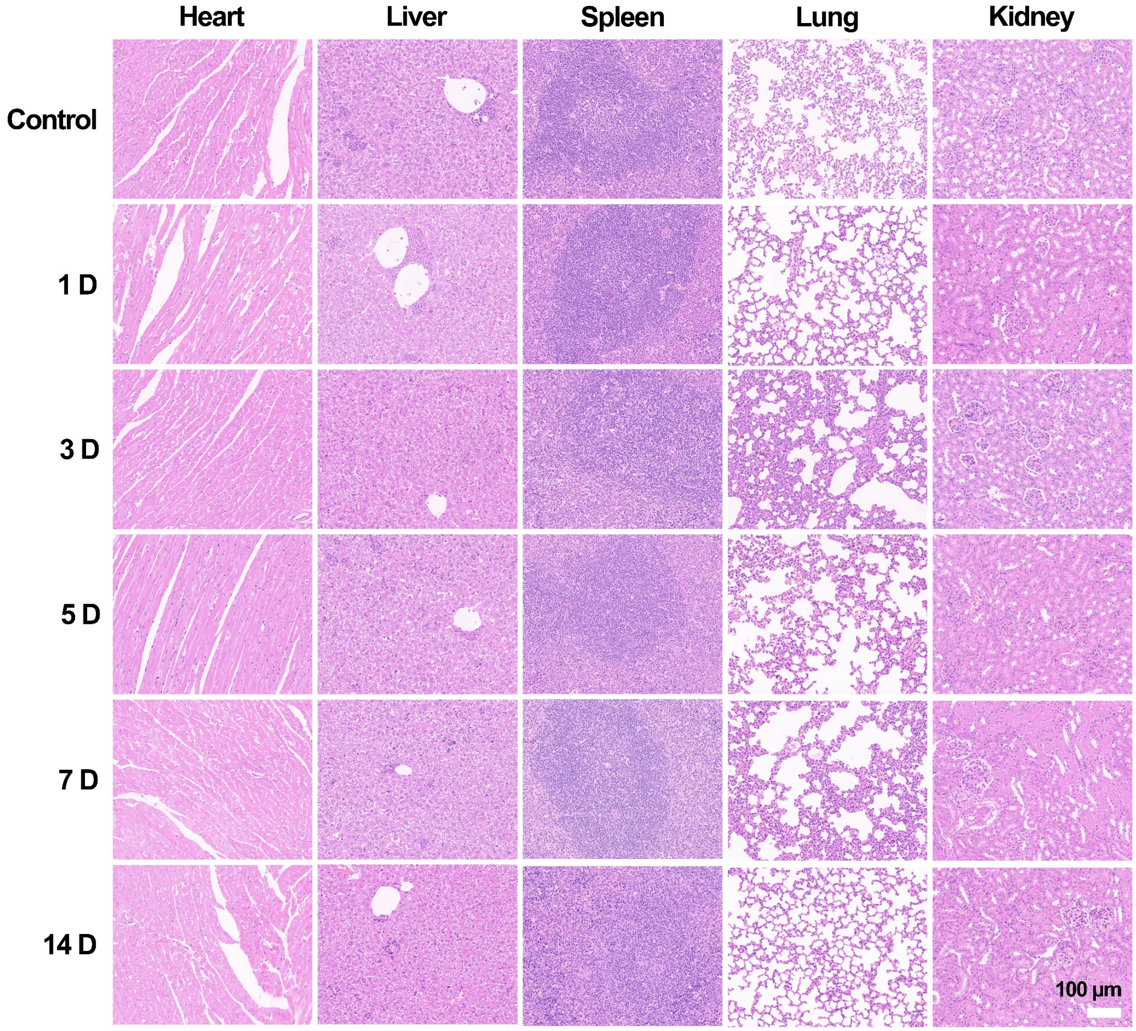

Figure S32. H&E staining images of major organs (heart, liver, spleen, lung, and kidney) of healthy Balb/c mice after intravenous injection of BiOBr@Bi_2S_3 (dose: 10 mg/kg) at different time points (1, 3, 5, 7, and 14 days).

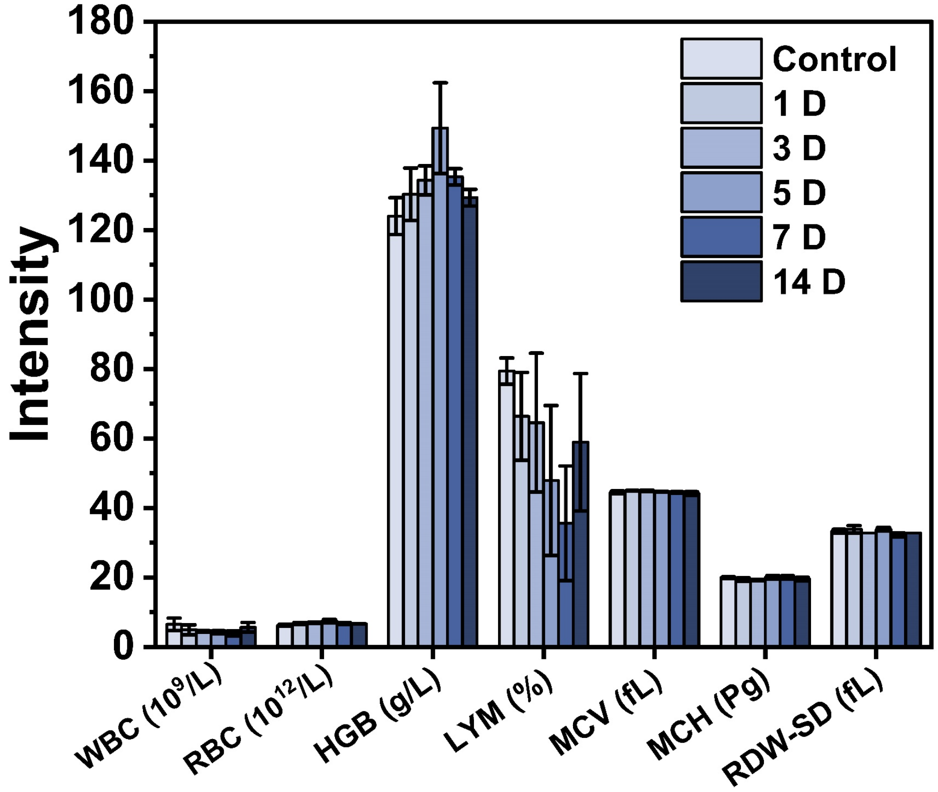

Figure S33. Blood hematological counts of the mice sacrificed at 1, 3, 5, 7, and 14 days after treatment with $BiOBr@Bi_2S_3$ (dose: 10 mg/kg). Blood levels of White blood cells (WBC), Red blood cells (RBC), Hemoglobin (HGB), Lymphocyte (LYM), Mean corpuscular volume (MCV), Mean corpuscular hemoglobin (MCH), and Red Cell volume Distribution Width SD (RDW-SD) (Mean ± SD, n = 3).

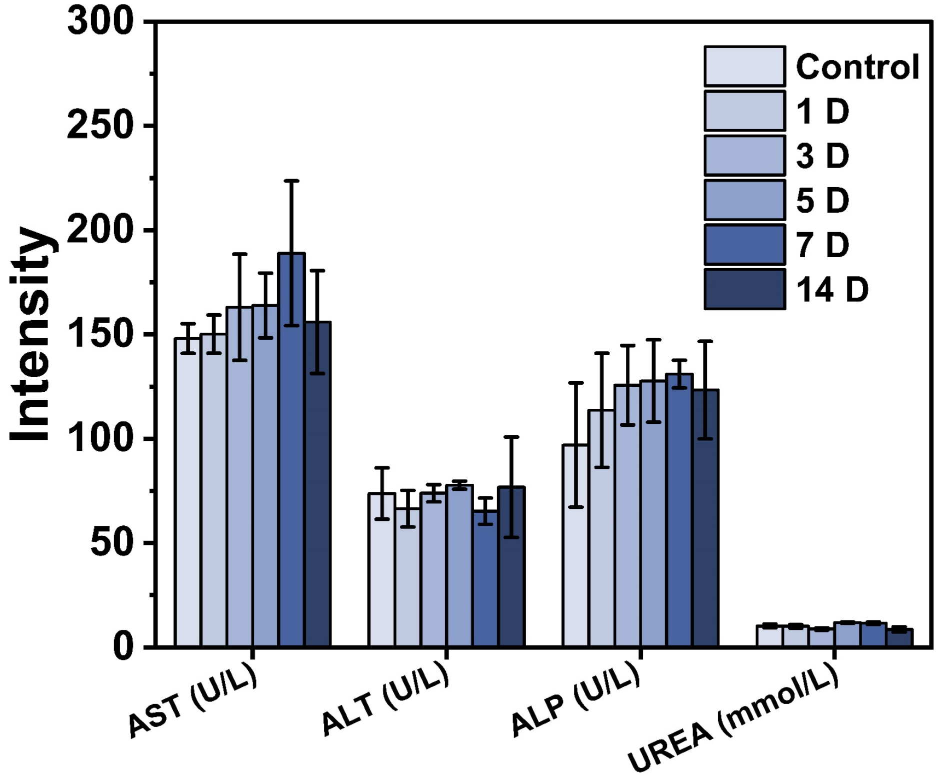

Figure S34. Serum biochemistry data of the mice sacrificed at 1, 3, 5, 7, and 14 days after treatment with BiOBr@Bi_2S_3 (dose: 10 mg/kg). Aspartate aminotransferase (AST), Alanine aminotransferase (ALT), and Alkaline phosphatase (ALP) as hepatic function indicators, and urea (UREA) as renal function indicators were measured (Mean ± SD, n = 3).

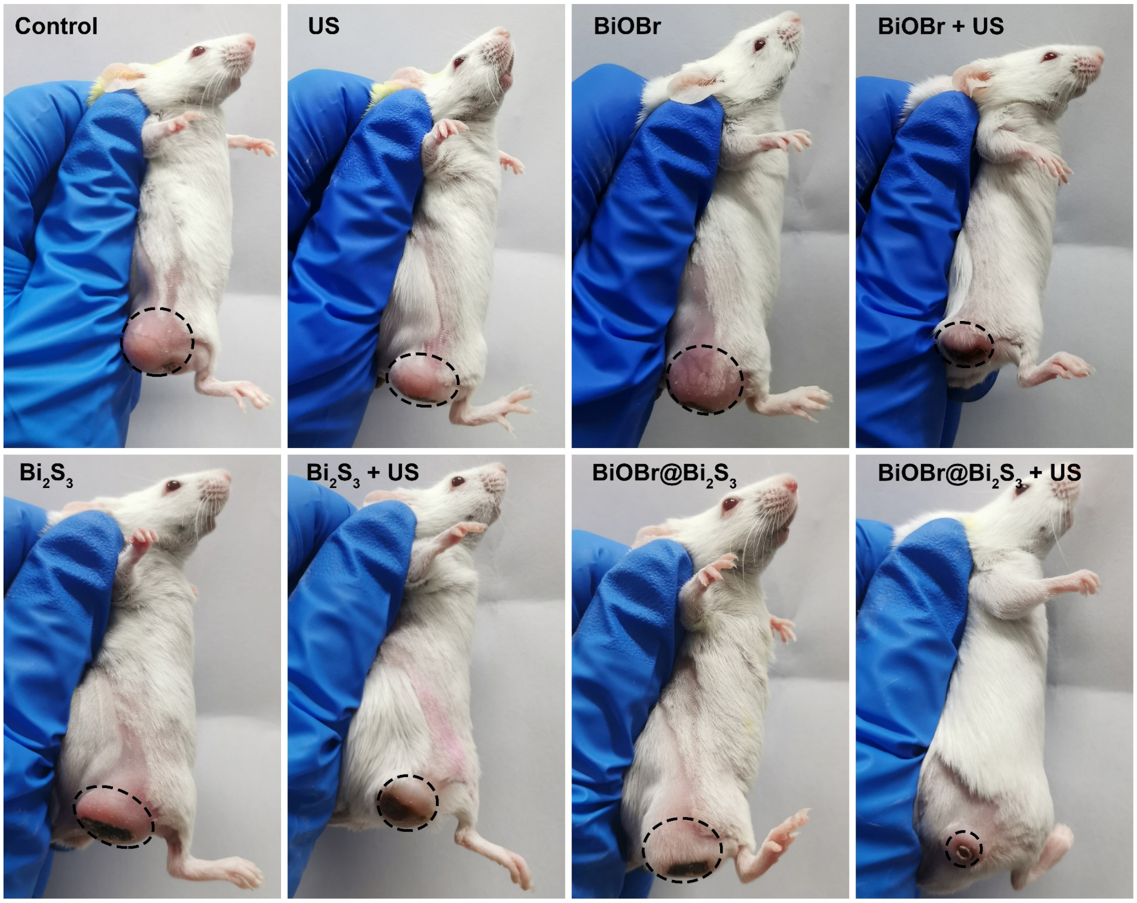

Figure S35. Photographs of mice in different treatment groups.

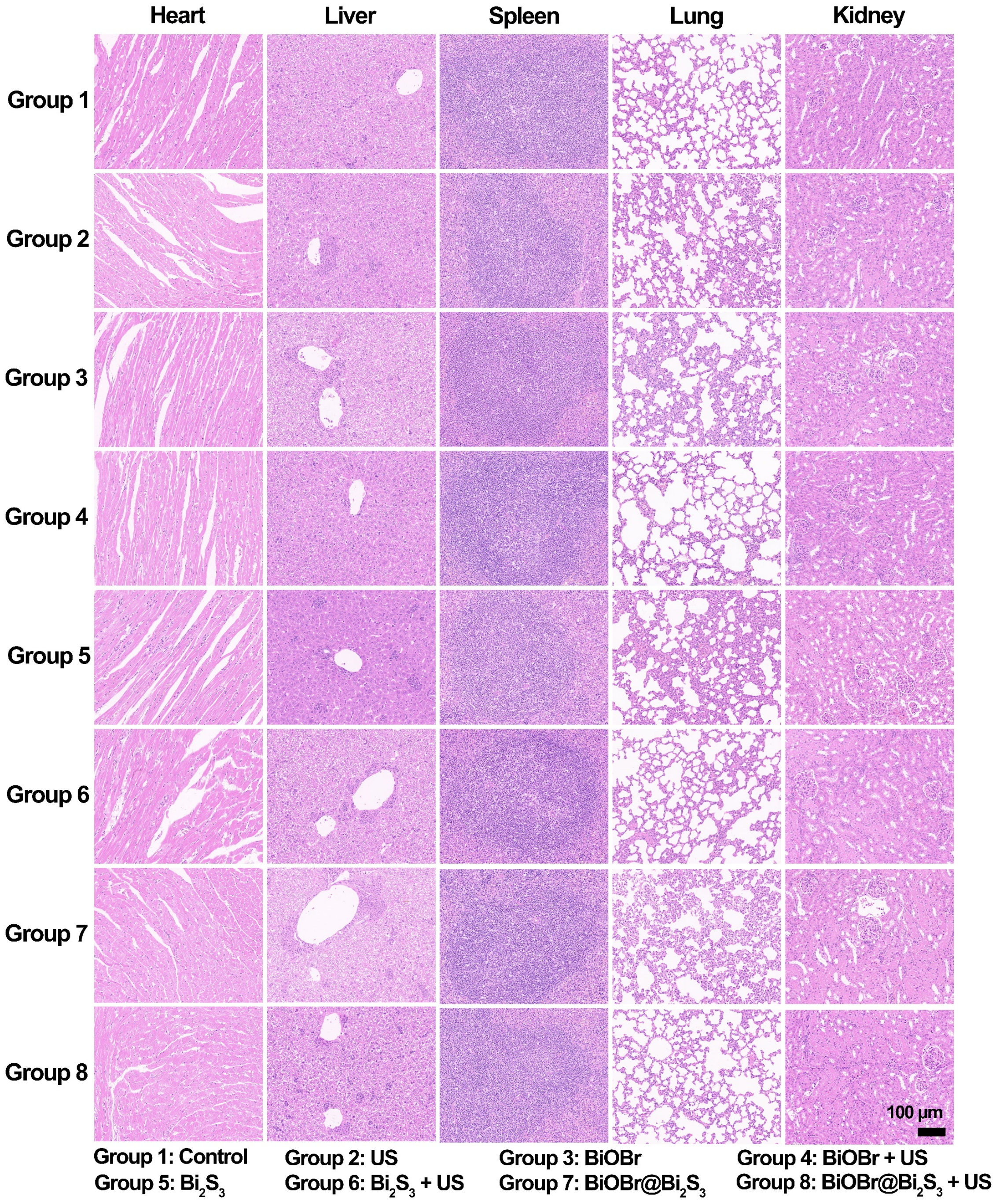

Figure S36. H&E staining images of main organs after different treatments.

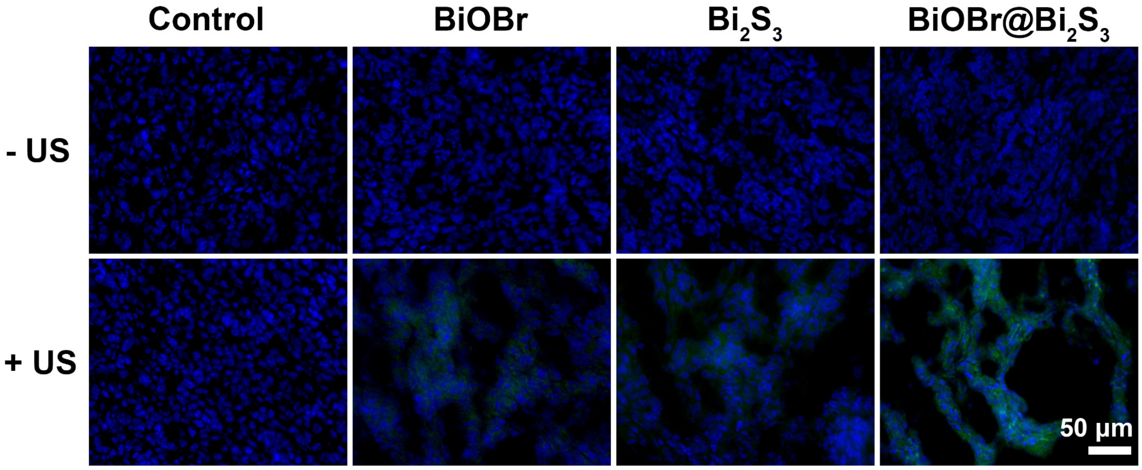

Figure S37. DCFH-DA staining images of tumor after different treatment.

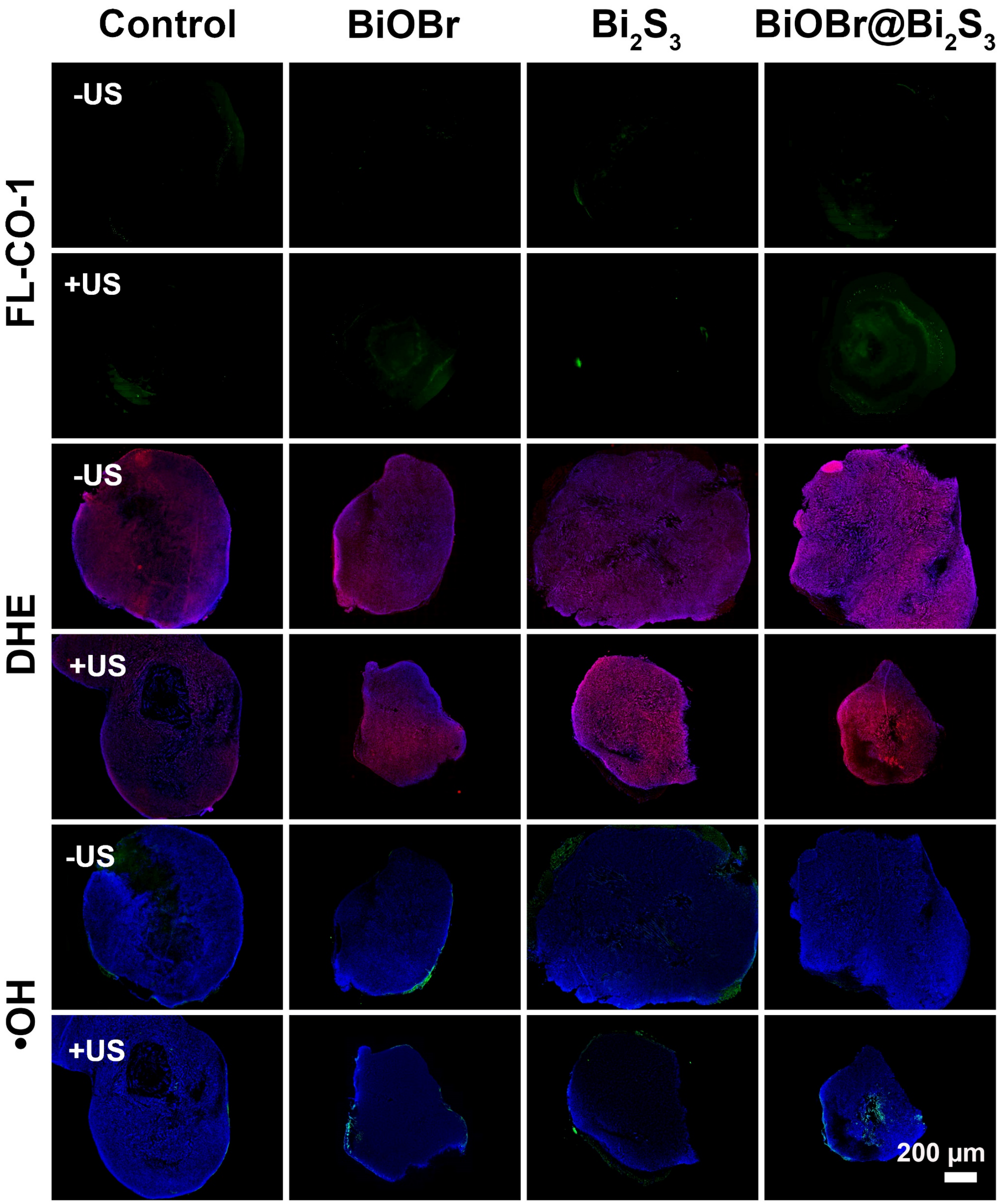

Figure S38. Immunofluorescent images of the tumors obtained from mice.

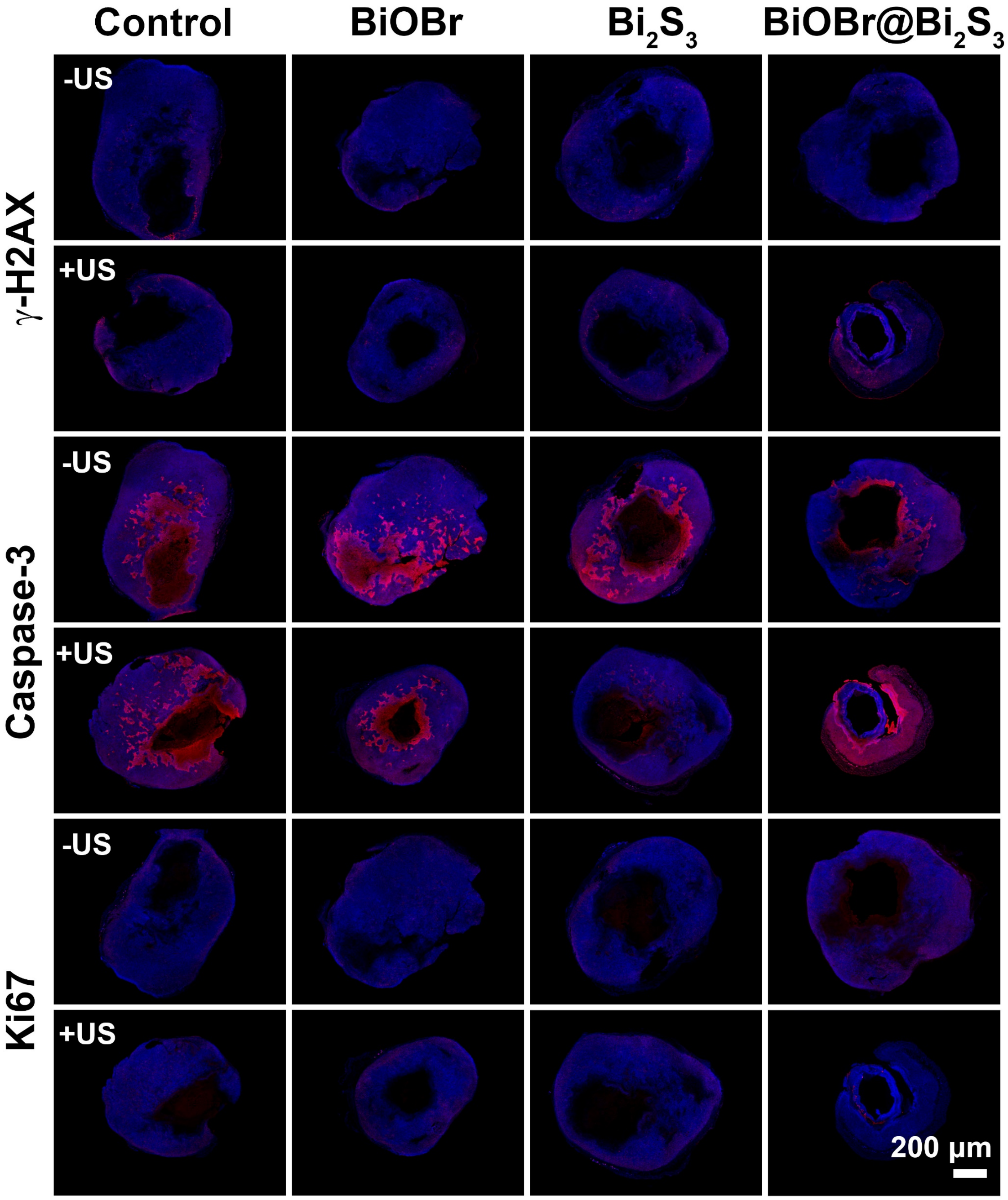

Figure S39. Immunofluorescent images of the tumors obtained from mice.

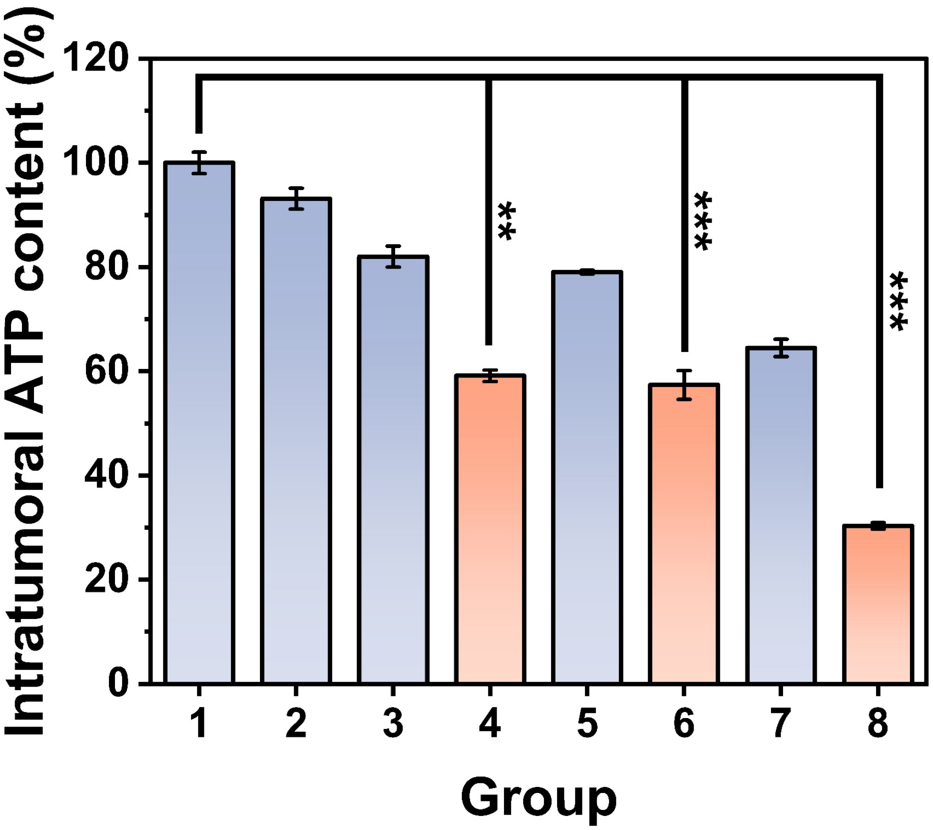

Figure S40. The intratumoral ATP level after different treatment (mean ± SD, n = 3). The *p* values were calculated by one-way analysis of variance (ANOVA), ****p < 0.0001, ***p < 0.001, **p < 0.01, and *p < 0.05. Group: 1) Control, 2) US, 3) BiOBr, 4) BiOBr + US, 5) Bi_2S_3, 6) Bi_2S_3 + US, 7) BiOBr@Bi_2S_3, 8) BiOBr@Bi_2S_3 + US.
